# Scanning of selection signature provides a glimpse into important economic traits in goats (*Capra hircus*)

**DOI:** 10.1038/srep36372

**Published:** 2016-10-31

**Authors:** Dailu Guan, Nanjian Luo, Xiaoshan Tan, Zhongquan Zhao, Yongfu Huang, Risu Na, Jiahua Zhang, Yongju Zhao

**Affiliations:** 1 College of Animal Science and Technology, Southwest University; Chongqing Key Laboratory of Forage & Herbivore; Chongqing Engineering Research Center for Herbivores Resource Protection and Utilization, Chongqing 400715, P. R. China

## Abstract

Goats (*Capra hircus*) are one of the oldest livestock domesticated species, and have been used for their milk, meat, hair and skins over much of the world. Detection of selection footprints in genomic regions can provide potential insights for understanding the genetic mechanism of specific phenotypic traits and better guide in animal breeding. The study presented here has generated 192.747G raw data and identified more than 5.03 million single-nucleotide polymorphisms (SNPs) and 334,151 Indels (insertions and deletions). In addition, we identified 155 and 294 candidate regions harboring 86 and 97 genes based on allele frequency differences in Dazu black goats (DBG) and Inner Mongolia cashmere goats (IMCG), respectively. Populations differentiation reflected by Fst values detected 368 putative selective sweep regions including 164 genes. The top 1% regions of both low heterozygosity and high genetic differentiation contained 239 (135 genes) and 176 (106 genes) candidate regions in DBG and IMCG, respectively. These genes were related to reproductive and productive traits, such as “neurohypophyseal hormone activity” and “adipocytokine signaling pathway”. These findings may be conducive to molecular breeding and the long-term preservation of the valuable genetic resources for this species.

There are over 300 distinct breeds of goat (*Capra hircus*) in the world, which are distributed over all types of ecological areas with more concentrated in the tropics, dry zones and developing countries. Goats have been used for their milk, meat, hair, or cashmere and skins over much of the world. In 2011, there were more than 1.4 billion live goats around the globe, according to the UN Food and Agriculture Organization (http://kids.fao.org/glipha/). China is rich in goat genetic resources, and there are about 69 goat breeds in this country, including 58 local breeds, 8 improved breeds and 3 introduced breeds. Especially, Inner Mongolia cashmere goat (IMCG), mainly lived in Alashan, Inner Mongolia, in the north of China (~1304 m) coated white long hair, which produced primarily cashmere and had relatively low fecundity (~105%) compared with Duzu black goat (DBG, ~272%), black short hair, which are raised in Dazu County of Chongqing in the southwest of China (267~934 m) and are primarily used for meat production^1^ (Fig. 1a).

Goat is one of the earliest animals domesticated by humans. The most recent genetic analysis confirms the archaeological evidence that the wild Bezoar ibex of the Zagros Mountains are the likely origin of almost all domestic goats today. But the morphologicaland behavioral characteristics of modern domestic goats have greatly changed compared with wild progenitor, the Bezoar goat (*Capra aegagrus*)^2^. Compared with the Bezoar goat, modern goats exhibit a reduction in body size, a more docile demeanor, a series of coat color variants and the ability to adapt after domestication and breed formation, which have left detectable signatures of selection within the genomes^3^,^4^. Hence, scanning of selection signature can’t only provide straightforward insights into the mechanism of evolution events but also use for identifying functional genes of important economic traits or causative variations of phenotypic diversity.

Based on populations differentiation reflected by Fst statistic, Gibbs *et al*. detected some genomic regions embracing some SNPs in genes associated with meat quality (*SPOCK1*) and feed efficiency (*ZRANB3, R3HDM1*)^5^. The differences in allele frequencies were correlated with the effects of those SNPs on various production traits. For instance, Xu *et al*. identified some of less-known genes under positive selection correlated with milk production traits such as *LAP3* and *SAR1B*, associated with hair length, namely, *FGF5*^6^, and related to reproduction traits such as *TSHR, GHR* and *BMP15*^7^,^8^. However, studies of selection signature on goats are still incipient compared with studies of other livestock. The goal of the present study was to identify a number of candidate genes for available to study based on allele frequency differences and population genetic differentiation.

## Results

### Sequencing

**Figure 1:**
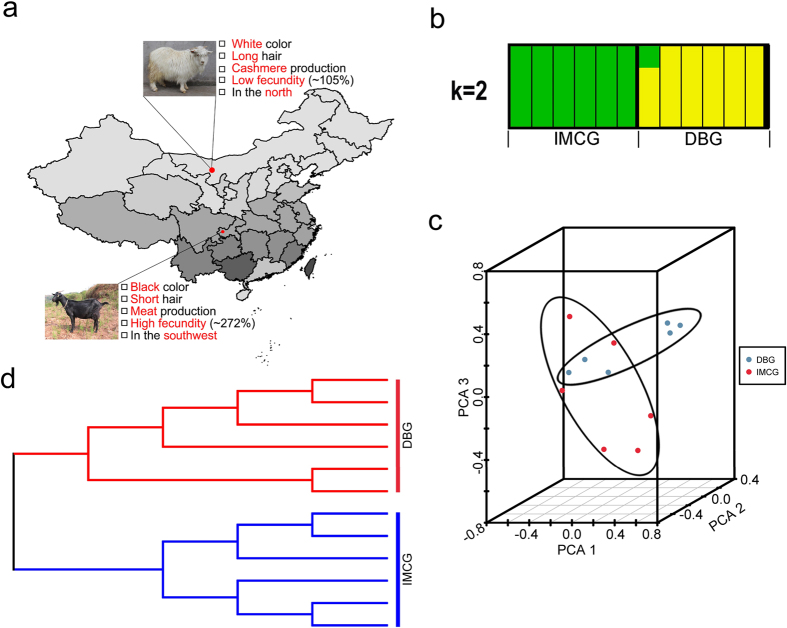
Samples distribution and population structure. (**a**) Distribution of goats sampled. The IMCG, which are white in color and are used in cashmere production, were from Alashan, Inner Mongolia, in the north of China (~1304 meters’ altitude). IMCG had relatively low fecundity (~105%) compared with DBG (~272%), which are primarily used for producing meat and are raised in Chongqing in the southwest of China (267~934 meters altitude). The geographic map was drawn by R software (https://www.r-project.org/). (**b**) PCA plot of two goat breeds. (**c**) Geneticstructure of two populations. Each color represents a cluster and each bar represents an individual. (**d**) NJ phylogenetic tree of 12 individuals. All pictures were modified by Adobe Photoshop CS5 (http://www.adobe.com/).

The genomes of 12 goats from two genetically diverse and geographically distinct indigenous breeds consists of DBG and IMCG (Fig. 1) were sequenced to average 6.02-fold coverage. A total of 192.74 G raw data generated 191.57 G clean data after a quality check and filtering by removing the adapter of paired reads or low quality reads. The effective rate and the ratio of clean data to raw data were 98.79% to 99.64%, respectively. We also measured the GC content distribution (45.06% of our data versus 41.75% of the reference sequence). Effective sequencing reads were aligned to CHIR_1.0 reference genome, resulting in ~100 million mapped reads. The mapping rate in different individuals were varies from 98.11% to 98.69%. The percentages of reads sequenced once or four times per bp were >97.8% and 62.72%, respectively (Supplementary Table 1).

### Variation discovery

After assembly and mapping of clean reads, we identified 5.03 million SNPs and 334,151 Indels (insertions and deletions) as average. Furthermore, the distributions of the SNPs were determined in the goat genome that there were 37,684 (0.75%) and 38,976 (0.77%) SNPs in the exon in DBG and IMCG, respectively, while the intronic regions contained 28.39% and 28.04% of the SNPs in DBG and IMCG, respectively. SNPs within intergenic regions accounted for up to 69.51% and 68.09% of the SNPs in DBG and IMCG, respectively (Table 1). The results indicated that similar heterozygosity rates existed between the two populations (1.1622 of the DBG population versus 1.1605 of the IMCG population). The number of SNPs in each category of mutations in the goat genome are shown in Table 2. As shown in Table 2, the ratio of transition (ts) to transversion (tv) was estimated to be 2.3347 in the DBG goats, slightly lower than that in the IMCG goats (2.3552). The detection of variation type showed that the classes of T: A > C: G or C: G > T: A were the primary types of variation (Supplementary Figure 1). In addition, the results showed that intergenic regions contained 66.44% of the Indels, intronic regions contained 30.31% of the Indels, and exons contained only 0.21% of the Indels (Table 1). The heterozygosity rate of Indels between the two goat populations was remarkably consistent (0.0602 of the DBG population versus 0.0600 of the IMCG population).

### Population structure analysis

Population substructure was investigated using Clustering, MEGA and ADMIXTURE softwares based on using genomic SNPs. We ran Admixture 1.22 for determining genetic backgrounds of samples and indicated two breeds been almost diverged but there is a little gene introgression in DBG when K = 2 (Fig. 1b). The results of principal component analysis (PCA) analysis showed that three principal components (PC1, PC2 and PC3) differentiated DBG and IMCG individuals (Fig. 1c). The neighbor-joining (NJ) tree confirmed these results and successfully divided into two populations displaying genetically distinct clusters (Fig. 1d). The clear genetic divergence between DBG and IMCG showed that the individuals chosen could be used for further exploring their genomic features.

### Analysis of Selective sweeps

**Figure 2:**
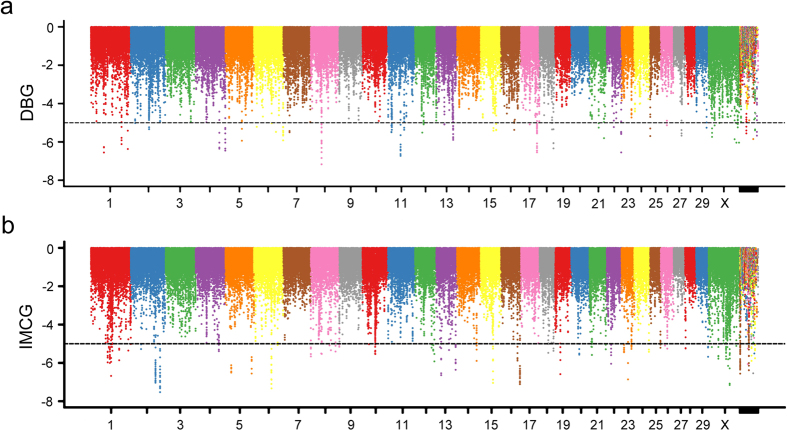
The Manhattan plot bsed on allele frequency differences in DBG breeds (**a**) and IMCG breeds (**b**).

**Figure 3:**
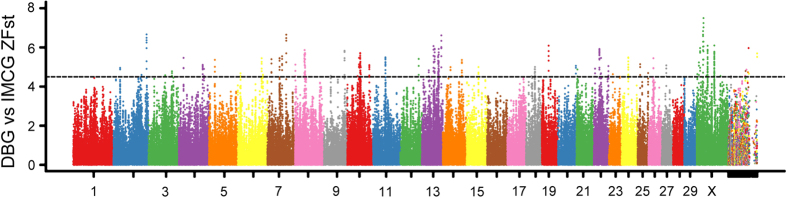
The distribution of Fst value across the genomes of the two goat breeds.

**Figure 4:**
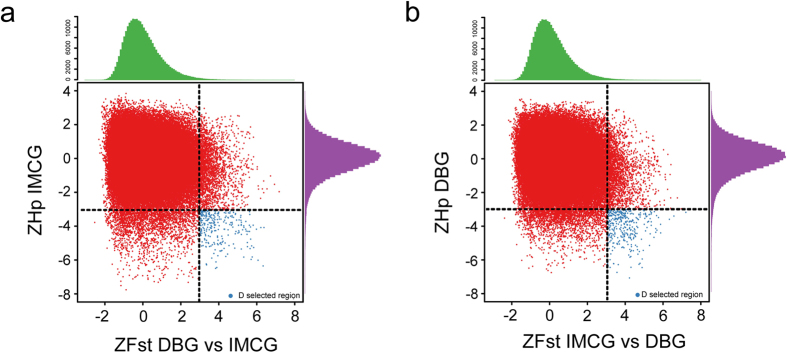
Identification of genomic regions with low heterozygosity and high genetic differentiation in DBG (**a**) and IMCG (**b**).

Those genomic regions with ZHp value exceeding the empirical threshold level of −5 across the two populations were obtained, that contain 155 and 294 candidate regions in the DBG and IMCG populations, respectively. The top regions were located on chromosome 8 in DBG goats (41890001–41950001 bp) and on chromosome 2 in IMCG goats (113850001–113900001 bp), respectively (Fig. 2a,b, Supplementary Table 2). To obtain the available candidate genes, all candidate regions were annotated using the first generation goat genome annotation information to identify candidate genes that underwent sweeps. A total of 86 and 97 genes in DBG goats and IMCG goats respectively were identified (Supplementary Table 5). Next, Fst values were measured, which a special statistic was used to detect the selection signature based on genetic differentiation that resulted from genetic drift and selective pressure. The result showed that a total of 368 putative selective sweep regions containing 164 candidate genes exceeding the empirical threshold level of 4.5 were obtained (Fig. 3, Supplementary Tables 3 and 5). To further explore the sweep regions, those regions that contained both low heterozygosity and high genetic differentiation were scanned and contained a total of 239 regions that ranked in the top 1% based on the |ZHp| and ZFst value in both groups. There were 239 and 176 outliers detected in the DBG and IMCG, respectively (Fig. 4a,b, Supplementary Table 4). There are 135 and 106 gene candidates in DBG and IMCG, respectively (Supplementary Table 5). Gene ontology (GO) and pathway analysis (KEGG) for candidate genes under selection were performed in order to further explore the functions of the selective sweep gene candidates in detail. All *P* *<* *0.05* GO terms and KEGG pathways were deposited in Supplementary Tables 6 and 7, respectively. In total, we annotated 386 GO entries (Supplementary Table 6) and 31 KEGG pathways (Supplementary Table 7).

### Candidates affecting goat reproduction

Reproduction trait is an important component of efficiency in goat production system^24^. A plurality of reproduction-related categories were identified, such as, (1) Hp values: “neurohypophyseal hormone activity” (3 genes, *PAIP2B, CCDC64, EPB41L5*), “photoreceptor activity” and “blue light photoreceptor activity” (*BIRC6*) in DBG goats; “meiosis” and “meiotic cell cycle” (4 genes, *TAOK1, C6H4orf22, SGOL1, SLC33A1*), “meiosis I” (3 genes, *SLC33A1, C6H4orf22, TAOK1*) in IMCG population; (2) Fst values: “sex determination” and “mating type determination” (2 genes, *PAIP2, CLEC16A*), “pheromone activity” (4 genes, *ZNF280D, CLEC16A, ARID1B, PAIP2*). (3) Combination |ZHp| with Fst values: “gamete generation” (8 genes, *SBF1, PRTG, PARD3B, KDM4C, FAT1, DMD, TPPP3, DACH2*), “neurohypophyseal hormone activity” (4 genes, *KDM4C, RYBP, FARP1, CELF2*),“spermatid development”(3 genes, *PARD3B*,*FAT1*,*KDM4C*), “spermatid nucleus differentiation”,“sperm chromatin condensation”, “spermatid differentiation” and “spermatid differentiation” (3 genes, *PARD3B, FAT1, KDM4C*), “spermatogenesis” and “male gamete generation” (5 genes, *FAT1, KDM4C, TPPP3, SBF1, PARD3B*) (Supplementary Tables 6 and 7).

### Candidates related to productive traits

The rumen, which is the largest compartment, encompasses numerous symbiotic microbial floras to ferment the feed for lipid metabolism^10^. We identified a total of 3 pathways, as followings: “adipocytokine signaling pathway” (2 genes, *IKBKG, LOC102190823*), “ether lipid metabolism” (2 genes, *PLD2, PLA2G1B*), “Glycosphingolipid biosynthesis-ganglio series” (*SLC33A1*), and 8 GO terms, such as, glycosphingolipid/galactolipid metabolic process (*LOC102171901*), galactolipid/glycolipid/sphingolipid/membrane lipid/glycosylceramide/glycosphingolipid catabolic process (*LOC102171901*) associated with putative lipid metabolic pathways. In addition, There were “Ras signaling pathway” (5 genes, *RASGRP2, RRAS, PLA2G1B, KDR, IKBKG*), “ubiquitin mediated proteolysis” (4 genes, *STUB1, MID2, LOC102174728, BIRC6, UBE3B*), “VEGF signaling pathway” (2 genes, *PXN, KDR*), “MAPK signaling pathway” (4 genes, *RASGRP2, RRAS, PTPN7, IKBKG*), “glutathione metabolism” (2 genes, *LOC102188087, IDH1*), “ribosome” (10 genes, *LOC102178382, RPLP0, LOC102181054, LOC102173128, MROH8, LOC102190542, LOC102175291, LOC102176839, LOC102176407, LOC102181499*), “RNA polymerase” (2 genes, *POLR3F, POLR2C*), “purine metabolism” (3 genes, *AK3, POLR3F, POLR2C*), “biosynthesis of amino acids” (2 genes, *PYCR1, LOC102171894*), “pyrimidine metabolism” (2 genes, *POLR3F, POLR2C*) and “metabolic pathways” (19 genes, *LOC102179840, PANK2, LOC102178836, COX2, COX3, CYTB, COX1, ATP6, NFS1, ND4L, ATP8, LOC102171894, GLDC, ND1, ND3, ND2, ND5, ND4, ND6*) (Supplementary Tables 6 and 7). Our study suggests that the genes included these terms or pathways may be most likely related to productive traits.

## Discussion

Selection, which appear to have left detectable signatures of selection within the animal genomes is a vital driving force of evolution. Since the dawn of agriculture, artificial selection has continuously added to the existing pool of phenotypic variation as same as natural selection fuels the generation of biodiversity on earth. The programs of selection signature have identified hundreds of regions or genes targeted by important traits that formed by natural or artificial selection inchicken^8^, cattle^6^, swine^11^ and sheep^12^. It’s essential to identify candidate genes of important economic traits used for further studies in goats.

Hair length is a characteristic trait in cashmere goats in comparison to meat and milk goats. The hair length on IMCG (14.92 ± 4.41 cm) used in this study is significantly longer than DBG (3.92 ± 0.76 cm). Hair growth was considered to be regulated by hairless gene, *FOXI3*^13^, hypertrichosis gene, *FGF13, Trps1, Sox9*^14^,^15^, and hair overgrowth gene, *ABCA5*^16^. However, natural long hair, well described angora phenotype, results from a regulator gene, *FGF5*, in many kinds of mammals^17^,^18^,^19^,^20^,^21^. Here *FGF5* have been included in the candidate genes sets based on low heterozygosity and high genetic differentiation in IMCG population (Fig. 4, Supplementary Table 5). We detected three synonymous mutations: g. 92103757C > T (rs653636435), g. 92111741A > T (rs672199363), g. 92124227A > T (rs665580959), and four mutations that were absent from goat dbSNP database, namely g. 92124086T > G, g. 92124131A > G, g. 92124134G > C and g. 92124199C > T within the *FGF5* exon sequence. Only g. 92124199G > C candidate mutation could lead to a serine-to-leucine transitionat the 224^th^ site in FGF5 amino acid sequence and need to further verification, although He *et al*. demonstrated that the expression of *FGF5s*, a mRNA alternative splicing of *FGF5*, probably act as main cause of increasing the length of cashmere fiber^22^.

The seasonal reproduction is one of the most conspicuous characters in goats, which is archived by alternation between long day and short day. Light information received by photoreceptor organ uniquely positioned to eye in mammals is transmitted to the pineal gland through the suprachiasmatic nucleus and then give rise to hormone secretion, especially melatonin^23^. Consequently, light is one of the important factors affecting goat reproduction, which is important component of efficiency in goat production system^24^. *BIRC6* gene acted as “photoreceptor activity” or “blue light photoreceptor activity” was obtained based on heterozygosity as a result of differences of annual insolation duration between two populations (IMCG, 3000–3400 h; DBG, 1279 h)^1^ (Supplementary Table 5). Previous research has reported polymorphisms in the *BIRC6* gene were associated with hereditary eye diseases, pseudoexfoliative glaucoma^25^. And the mutations of *BIRC6* would result in abnormal embryonic development^26^,^27^. Walsh *et al*. determined blue light was indeed associated with melatonin expression in horses^28^. Goat reproductive traits are the result of the combined effects of multiple organ, such as, the above-mentioned photoreceptor uniquely positioned to eye, pituitary and even olfactory. The male goats secret pheromone, which is received by the pheromone receptor located at the olfactory epithelium or the vomeronasal organ. The pheromone signal is integrated and then induce hypothalamic-pituitary hormone cascade, finally induce an out-of-seasonal ovulation in anoestrous females, so-called “male effect”^29^,^30^. Two terms, “neurohypophyseal hormone activity” and “pheromone activity” (Supplementary Table 6) may probably illustrate the mechanism of this effect. The complement of these analyses may therefore accelerate the pace of improvements of goat genetics.

Goats are browsing animals, digesting crude fiber in a four-chambered stomach compared with monogastric livestock, for example swine. The rumen engaged in lipid metabolism implemented by numerous symbiotic microbialfloras^31^. The level of lipid metabolism is closely related to fat deposition, such as *IKBKG*^9^*, LOC102190823* (Supplementary Table 5); muscle mass for example *PLD2*^10^ (Supplementary Table 5); and milk production^32^. *IDH1* gene (Supplementary Table 5), including in the term, “glutathione metabolism” (Supplementary Table 7) is significantly associated with increased milk citrate content^33^. Dissecting major-genes of production traits are difficult only depending on sequencing data. The results provided by this study can provide some routes for future researches.

In summary, the study presented here has generated 192.747 G raw data based on whole-genome re-sequencing. A total of more than 5.03 million SNPs and 334,151 Indels were identified by resequencing 12 goat samples. The selective sweep analysis revealed some interesting candidate genes and pathways affected by natural or artificial selection involving reproductive or productive traits. The works performed here provided an important resource for future quantitative trait loci mapping of the goat breeding.

## Materials and Methods

### Experimental samples

This study was carried out in strict accordance with the recommendations in the Guide for the International Cooperation Committee of Animal Welfare (ICCAW), which is responsible for animal care and use in China. The experimental conditions were approved by the Committee on the Ethics of Animal Experiments of Southwest University (No. [2007] 3). All experimental goats were fed a daily ration of 300–500 gconcentrate and were allowed to an unrestricted access to straw, mineral saltlick and water.

We collected a total of 12 blood samples of unrelated individuals from 6 Dazu black female goats (DBG) and 6 Inner Mongolia cashmere female goats (IMCG) (Fig. 1a). All the goats were housed in Dazu Black Goat Farm at Southwest University, Chongqing, China. Genomic DNA was extracted using a Tiangen DNA isolation kit (Tiangen Biotech, Beijing, China). In addition, we calculated the average hair length of 20 IMCG and 5 DBG, respectively with software SPSS 19.0.

### Sequencing and variation calling

Each DNA sample was used to construct 350 bp paired-end sequencing libraries. Each library was sequenced with Illumina HiSeq PE150 platform according to the manufacturer’s instructions at Novogene (Tianjin, China). Raw data were processed to filter out the adaptors and low quality reads resulted in clean reads using BWA software^34^. After alignment, variation calling was performed per individual using SAMtools package^35^. All the called variants were annotated using the ANNOVAR package with the gene-based, or region-based, or filter-based optionsfor further analysis^36^.

### Population structure analysis

We used the Cluster 3.0 software for performing PCA analysis with the population scale SNPs^37^,^38^. Genetic structure was estimated using the program ADMIXTURE Version 1.22^39^, which was run at K = 2 on the basis of the whole SNP dataset. The neighbor-joining (NJ) tree was constructed using MEGA 5.2 procedure based on genome-wide SNPs^40^,^41^.

### Analysis of selective sweep

Selective sweep analyses were performed by calculating heterozygosity (Hp) and population differentiation (Fst). The Hp and Fst values, respectively were converted to a standard normal distribution, denoted as ZHp and ZFst as described^42^,^43^. In addition, those regions that have low ZHp value and high ZFst value were scanned as candidates. To know the biological function of genes within candidate regions, the analysis of Gene Ontology (GO) and Kyoto Encyclopedia of Genes and Genomes (KEGG) pathways were performed using Goseq (Bioconductor 2.12) and KOBAS (kobas2.0–20120208), respectively.

## Additional Information

**How to cite this article**: Guan, D. *et al*. Scanning of selection signature provides a glimpse into important economic traits in goats (*Capra hircus*). *Sci. Rep.* **6**, 36372; doi: 10.1038/srep36372 (2016).

**Publisher’s note:** Springer Nature remains neutral with regard to jurisdictional claims in published maps and institutional affiliations.

## Supplementary Material

Supplementary Information

## Figures and Tables

**Table 1:** Characteristics of SNPs and indels identified in the two study cohorts.

Variationtype	Population	Exonic	Intronic	Intergenic	Total
SNPs	DBG	37,684	1,414,555	3,463,597	4,915,836
	IMCG	38,976	1,424,565	3,458,726	4,922,267
Indels	DBG	710	100,638	221,103	322,451
	IMCG	702	100,493	219,789	320,984

**Table 2:** The number of SNPs in the genomes of the two goat breeds.

	SG	SL	Syno.	Non-Syno.	Sp	Ts	Tv	Total
DBG	191	32	19,590	17,871	186	348,832	1,494,208	1,880,910
IMCG	204	34	20,348	18,390	205	356,865	1,513,718	1,909,764

SG, SL, Syno., Non-Syno., Sp, ts and tvrepresent stop gain, stop loss, synonymous, non-synonymous, splicings, transitions and transversions, respectively. Stop gain means that mutations make the gene gain termination codon. Stop loss means that mutations make the gene lose termination codon. Splicings mean mutation is located at the exon/intron border of 2 KB in the intron.

## References

[b1] Zhang B. . Animal genetics resources in China, sheep and goat, China National Commission of Animal Genetic Resources edn (China Agricultural Press, 2011).

[b2] Zeder M. A. & Hesse B. The initial domestication of goats (*Capra hircus*) in the Zagros mountains 10,000 years ago. Science 287, 2254–2257, doi: 10.1126/science.287.5461.2254 (2000). 10731145

[b3] Benjelloun B. . Characterizing neutral genomic diversity and selection signatures in indigenous populations of Moroccan goats (*Capra hircus*) using WGS data. Frontiers in Genetics 6, doi: 10.3389/fgene.2015.00107 (2015). PMC4387958 25904931

[b4] Dong Y. . Reference genome of wild goat (*Capra aegagrus*) and sequencing of goat breeds provide insight into genic basis of goat domestication. Bmc Genomics 16, doi: 10.1186/S12864-015-1606-1 (2015). PMC4455334 26044654

[b5] Gibbs R. A. . Genome-Wide Survey of SNP Variation Uncovers the Genetic Structure of Cattle Breeds. Science 324, 528–532, doi: 10.1126/science.1167936 (2009). 19390050 PMC2735092

[b6] Xu L. Y. . Genomic Signatures Reveal New Evidences for Selection of Important Traits in Domestic Cattle. Mol Biol Evol. 32, 711–725, doi: 10.1093/molbev/msu333 (2015). 25431480 PMC4441790

[b7] Zhu C. Y. . Detection of Selection Signatures on the X Chromosome in Three Sheep Breeds. Int J Mol Sci. 16, 20360–20374, doi: 10.3390/ijms160920360 (2015). 26343642 PMC4613208

[b8] Rubin C. J. . Whole-genome resequencing reveals loci under selection during chicken domestication. Nature 464, 587–U145, doi: 10.1038/nature08832 (2010). 20220755

[b9] Zhu L. . Distinct expression patterns of genes associated with muscle growth and adipose deposition in tibetan pigs: a possible adaptive mechanism for high altitude conditions. High Alt Med Biol. 10, 45–55, doi: 10.1089/ham.2008.1042 (2009). 19278352

[b10] Bernard C., Cassar-Malek I., Renand G. & Hocquette J. F. Changes in muscle gene expression related to metabolism according to growth potential in young bulls. Meat Sci. 82, 205–212, doi: 10.1016/j.meatsci.2009.01.012 (2009). 20416758

[b11] Li M. Z. . Whole-genome sequencing of Berkshire (European native pig) provides insights into its origin and domestication. Sci Rep-Uk 4, doi: 10.1038/Srep04678 (2014). PMC3985078 24728479

[b12] Manunza A. . Population structure of eleven Spanish ovine breeds and detection of selective sweeps with BayeScan and hapFLK. Sci Rep-Uk 6, 27296, doi: 10.1038/srep27296 (2016). PMC4895181 27272025

[b13] Drogemuller C. . A mutation in hairless dogs implicates *FOXI3* in ectodermal development. Science 321, 1462, doi: 10.1126/science.1162525 (2008). 18787161

[b14] Fantauzzo K. A., Kurban M., Levy B. & Christiano A. M. *Trps1* and Its Target Gene *Sox9* Regulate Epithelial Proliferation in the Developing Hair Follicle and Are Associated with Hypertrichosis. Plos Genet 8, doi: 10.1371/journal.pgen.1003002 (2012). PMC3486859 23133399

[b15] DeStefano G. M. . Position effect on *FGF13* associated with X-linked congenital generalized hypertrichosis. P Natl Acad Sci USA 110, 7790–7795, doi: 10.1073/pnas.1216412110 (2013). PMC3651487 23603273

[b16] DeStefano G. M. . Mutations in the Cholesterol Transporter Gene ABCA5 Are Associated with Excessive Hair Overgrowth. Plos Genetics. 10 (2014). 10.1371/journal.pgen.1004333 PMC4022463 24831815

[b17] DeStefano G. M. . Mutations in the Cholesterol Transporter Gene *ABCA5* Are Associated with Excessive Hair Overgrowth. Plos Genet 10, doi: 10.1371/journal.pgen.1004333 (2014). PMC4022463 24831815

[b18] Dierks C., Momke S., Philipp U. & Distl O. Allelic heterogeneity of *FGF5* mutations causes the long-hair phenotype in dogs. Anim Genet 44, 425–431, doi: 10.1111/age.12010 (2013). 23384345

[b19] Drogemuller C., Rufenacht S., Wichert B. & Leeb T. Mutations within the *FGF5* gene are associated with hair length in cats. Anim Genet 38, 218–221, doi: 10.1111/j.1365-2052.2007.01590.x (2007). 17433015

[b20] Hebert J. M., Rosenquist T., Gotz J. & Martin G. R. *Fgf5* as a Regulator Of the Hair-Growth Cycle - Evidence From Targeted And Spontaneous Mutations. Cell 78, 1017–1025, doi: 10.1016/0092-8674(94)90276-3 (1994). 7923352

[b21] Legrand R., Tiret L. & Abitbol M. Two recessive mutations in *FGF5* are associated with the long-hair phenotype in donkeys. Genet Sel Evol 46, doi: 10.1186/s12711-014-0065-5 (2014). PMC4175617 25927731

[b22] He X., Chao Y., Zhou G. & Chen Y. Fibroblast growth factor 5-short (*FGF5s*) inhibits the activity of *FGF5* in primary and secondary hair follicle dermal papilla cells of cashmere goats. Gene 575, 393–398, doi: 10.1016/j.gene.2015.09.034 (2016). 26390813

[b23] Nakane Y. & Yoshimura T. Universality and diversity in the signal transduction pathway that regulates seasonal reproduction in vertebrates. Front Neurosci-Switz 8, doi: 10.3389/Fnins.2014.00115 (2014). PMC4033074 24959116

[b24] Ahlawat S., Sharma R., Maitra A. & Tantia M. S. Current status of molecular genetics research of goat fecundity. Small Ruminant Res. 125, 34–42, doi: 10.1016/j.smallrumres.2015.01.027 (2015).

[b25] Ayub H. . Association of a Polymorphism in the *BIRC6* Gene with Pseudoexfoliative Glaucoma. Plos One 9, doi: 10.1371/journal.pone.0105023 (2014). PMC4132048 25118708

[b26] Hitz C., Vogt-Weisenhorn D., Ruiz P., Wurst W. & Floss T. Progressive loss of the spongiotrophoblast layer of *Birc6*/*Bruce* mutants results in embryonic lethality. Genesis 42, 91–103, doi: 10.1002/gene.20128 (2005). 15887267

[b27] Salilew-Wondim D. . Depletion of *BIRC6* leads to retarded bovine early embryonic development and blastocyst formation *in vitro*. Reprod Fert Develop. 22, 564–579, doi: 10.1071/RD09112 (2010). 20188030

[b28] Walsh C. M., Prendergast R. L., Sheridan J. T. & Murphy B. A. Blue light from light-emitting diodes directed at a single eye elicits a dose-dependent suppression of melatonin in horses. Vet J 196, 231–235, doi: 10.1016/j.tvjl.2012.09.003 (2013). 23079244

[b29] Murata K. . Identification of an Olfactory Signal Molecule that Activates the Central Regulator of Reproduction in Goats. Curr Biol. 24, 681–686, doi: 10.1016/j.cub.2014.01.073 (2014). 24583018

[b30] Murata K. . Modulation of Gonadotrophin-Releasing Hormone Pulse Generator Activity by the Pheromone in Small Ruminants. J Neuroendocrinol. 21, 346–350, doi: 10.1111/j.1365-2826.2009.01836.x (2009). 19207811

[b31] Jiang Y. . The sheep genome illuminates biology of the rumen and lipid metabolism. Science 344, 1168–1173, doi: 10.1126/science.1252806 (2014). 24904168 PMC4157056

[b32] Castillo-Lopez E. . Ration formulations containing reduced-fat dried distillers grains with solubles and their effect on lactation performance, rumen fermentation, and intestinal flow of microbial nitrogen in Holstein cows. J Dairy Sci. 97, 1578–1593, doi: 10.3168/jds.2013-6865 (2014). 24440246

[b33] Canovas A. . RNA sequencing to study gene expression and single nucleotide polymorphism variation associated with citrate content in cow milk. J Dairy Sci. 96, 2637–2648, doi: 10.3168/jds.2012-6213 (2013). 23403202

[b34] Li H. & Durbin R. Fast and accurate short read alignment with Burrows-Wheeler transform. Bioinformatics 25, 1754–1760, doi: 10.1093/bioinformatics/btp324 (2009). 19451168 PMC2705234

[b35] Li H. . The Sequence Alignment/Map format and SAMtools. Bioinformatics 25, 2078–2079, doi: 10.1093/bioinformatics/btp352 (2009). 19505943 PMC2723002

[b36] Wang K., Li M. Y. & Hakonarson H. ANNOVAR: functional annotation of genetic variants from high-throughput sequencing data. Nucleic Acids Res. 38, doi: 10.1093/nar/gkq603 (2010). PMC2938201 20601685

[b37] Dunteman G. H. *Principal components analysis*. (Sage, 1989).

[b38] de Hoon M. J., Imoto S., Nolan J. & Miyano S. Open source clustering software. Bioinformatics 20, 1453–1454, doi: 10.1093/bioinformatics/bth078 (2004). 14871861

[b39] Alexander D. H., Novembre J. & Lange K. Fast model-based estimation of ancestry in unrelated individuals. Genome research 19, 1655–1664, doi: 10.1101/gr.094052.109 (2009). 19648217 PMC2752134

[b40] Saitou N. & Nei M. The neighbor-joining method: a new method for reconstructing phylogenetic trees. Mol Biol Evol. 4, 406–425 (1987). 3447015 10.1093/oxfordjournals.molbev.a040454

[b41] Tamura K. . MEGA5: Molecular Evolutionary Genetics Analysis Using Maximum Likelihood, Evolutionary Distance, and Maximum Parsimony Methods. Mol Biol Evol. 28, 2731–2739, doi: 10.1093/molbev/msr121 (2011). 21546353 PMC3203626

[b42] Rubin C. J. . Strong signatures of selection in the domestic pig genome. P Natl Acad Sci USA 109, 19529–19536, doi: 10.1073/pnas.1217149109 (2012). PMC3511700 23151514

[b43] Weir B. S. & Cockerham C. C. Estimating F-Statistics for the Analysis Of Population-Structure. Evolution 38, 1358–1370, doi: 10.2307/2408641 (1984). 28563791

