# Supplementary material for: Scanning of selection signature provides a glimpse into important economic traits in goats (Capra hircus)
Source: Sci Rep. 2016 Oct 31;6:36372. doi: 10.1038/srep36372 (PMC5087083; doi:10.1038/srep36372)
Supplement: Supplementary Information [file srep36372-s1.pdf]

Scanning of selection signature provides a glimpse into important economic traits in goats (*Capra hircus*)

Dailu Guan[1], Nanjian Luo[1], Xiaoshan Tan[1], Zhongquan Zhao[1], Yongfu Huang[1], Risu Na[1], Jiahua Zhang[1], Yongju Zhao[1]*

[1]College of Animal Science and Technology, Southwest University; Chongqing Key Laboratory of Forage & Herbivore; Chongqing Engineering Research Center for Herbivores Resource Protection and Utilization, Chongqing 400715, P. R. China

[*]Corresponding. zyongju@163.com

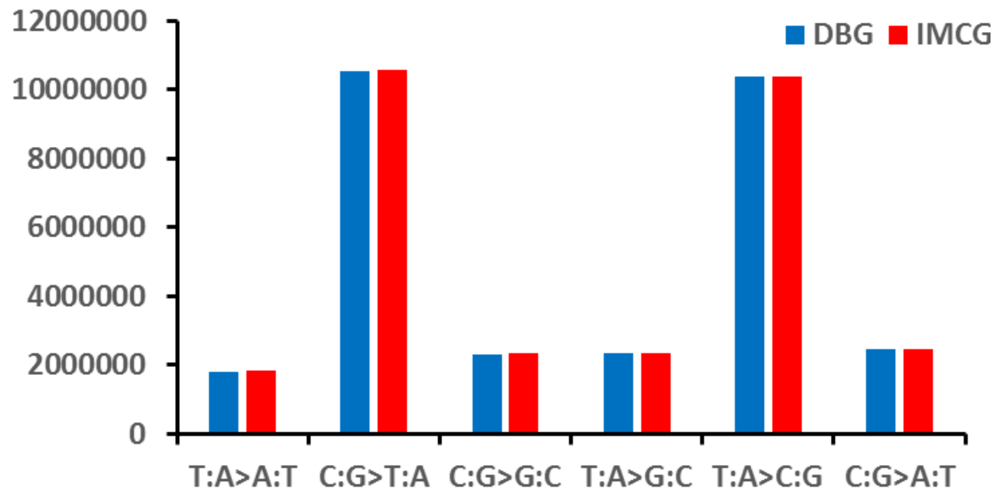

Supplementary figure 1 The distribution of the SNP variation types

Supplementary table 1 Summary statistics for the whole-genome resequencing

	DBG	IMCG
Number	6	6
Raw data	101,663,512,200	96,062,882,100
clean data	101,174,030,700	95,357,298,300
Mapped reads	663,948,838	625,085,291
Effective Rate(%)	99.52	99.27
Mapping rate(%)	98.44	98.33
GC Content(%)	45.11	45.02
Average depth(×)	6.16	5.88
1×Coverage (%)	96.73	96.45
4×Coverage (%)	70.03	67.40

Supplementary table 2 Candidate regions of selective sweep based on alleles frequency difference.

DBG				IMCG			
CHROM	POS	HP	ZHP<-5	CHROM	POS	HP	ZHP<-5
NC_022300.1	41890001	0.01	-7.17	NC_022294.1	1.14E+08	0.01	-7.53
NC_022300.1	41900001	0.02	-6.84	NC_022298.1	68060001	0.02	-7.33
NC_022303.1	48800001	0.03	-6.72	NC_022294.1	1.14E+08	0.02	-7.33
NC_022303.1	48780001	0.03	-6.63	NC_022294.1	1.14E+08	0.02	-7.26
NC_022293.1	50360001	0.03	-6.55	NC_022294.1	1.14E+08	0.02	-7.22
NC_022314.1	56530001	0.03	-6.55	NC_022322.1	82180001	0.03	-7.15
NC_022309.1	62150001	0.03	-6.54	NC_022308.1	73520001	0.03	-7.11
NC_022300.1	41880001	0.04	-6.53	NC_022298.1	67630001	0.03	-7.10
NC_022309.1	62160001	0.04	-6.45	NC_022322.1	82190001	0.03	-7.08

NC_022303.1	48810001	0.04	-6.45	NC_022307.1	47610001	0.03	-7.04
NC_022296.1	1.15E+08	0.04	-6.45	NC_022294.1	95830001	0.03	-7.03
NC_022303.1	48790001	0.04	-6.39	NC_022308.1	73510001	0.03	-7.03
NC_022293.1	1.43E+08	0.04	-6.37	NC_022294.1	1.14E+08	0.03	-7.00
NC_022296.1	92030001	0.04	-6.35	NC_022294.1	95820001	0.03	-6.99
NC_022296.1	1.15E+08	0.04	-6.35	NC_022308.1	73470001	0.03	-6.96
NC_022310.1	54440001	0.04	-6.34	NC_022294.1	95800001	0.04	-6.94
NC_022309.1	62130001	0.04	-6.32	NC_022294.1	1.14E+08	0.04	-6.93
NC_022309.1	62140001	0.05	-6.31	NC_022294.1	96080001	0.04	-6.93
NC_022300.1	41910001	0.05	-6.31	NC_022294.1	95810001	0.04	-6.90
NC_022296.1	92040001	0.05	-6.29	NC_022298.1	68070001	0.04	-6.88
NC_022293.1	50370001	0.05	-6.29	NC_022307.1	47620001	0.04	-6.87
NC_022293.1	50380001	0.05	-6.28	NC_022315.1	25100001	0.04	-6.86
NC_022293.1	50350001	0.05	-6.26	NC_022298.1	67620001	0.04	-6.85
NC_022303.1	48820001	0.05	-6.26	NC_022308.1	73490001	0.04	-6.80
NC_022309.1	62070001	0.05	-6.22	NC_022294.1	96070001	0.04	-6.80
NC_022303.1	48830001	0.05	-6.13	NC_022308.1	73480001	0.04	-6.73
NC_022293.1	1.19E+08	0.05	-6.11	NC_022294.1	1.14E+08	0.04	-6.73
NC_022300.1	41920001	0.06	-6.08	NC_022294.1	96010001	0.05	-6.69
NC_022309.1	62170001	0.06	-6.08	NC_022293.1	78110001	0.05	-6.68
NC_022310.1	54430001	0.06	-6.07	NC_022294.1	1.14E+08	0.05	-6.66
NC_022310.1	54420001	0.06	-6.06	NC_022305.1	18870001	0.05	-6.64
NC_022296.1	1.15E+08	0.06	-6.04	NC_022308.1	73460001	0.05	-6.62
NC_022322.1	1.08E+08	0.06	-6.04	NC_022294.1	96060001	0.05	-6.62
NC_022322.1	1.18E+08	0.06	-6.04	NC_022311.1	20040001	0.05	-6.59
NC_022296.1	92050001	0.06	-6.04	NC_022294.1	96030001	0.05	-6.58
NC_022300.1	42000001	0.06	-6.00	NW_005101032.1	160001	0.05	-6.56
NC_022297.1	63960001	0.06	-5.94	NC_022308.1	73550001	0.05	-6.55
NC_022300.1	41870001	0.06	-5.92	NC_022297.1	1.02E+08	0.05	-6.55
NC_022298.1	1.13E+08	0.06	-5.92	NC_022305.1	18880001	0.05	-6.54
NC_022293.1	1.19E+08	0.06	-5.91	NW_005101872.1	40001	0.05	-6.54
NC_022305.1	65530001	0.06	-5.89	NC_022308.1	73500001	0.05	-6.54
NC_022296.1	1.15E+08	0.07	-5.86	NC_022294.1	95850001	0.05	-6.53
NC_022322.1	1.01E+08	0.07	-5.86	NC_022294.1	1.14E+08	0.05	-6.52
NW_005101872.1	40001	0.07	-5.85	NC_022308.1	73540001	0.05	-6.51
NC_022313.1	56530001	0.07	-5.80	NC_022298.1	67650001	0.05	-6.50
NC_022305.1	65540001	0.07	-5.80	NC_022297.1	22420001	0.05	-6.50
NC_022293.1	1.19E+08	0.07	-5.79	NC_022294.1	96050001	0.05	-6.50
NC_022305.1	65520001	0.07	-5.75	NC_022294.1	95840001	0.06	-6.48
NC_022322.1	13450001	0.07	-5.73	NC_022297.1	22430001	0.06	-6.45
NC_022317.1	520001	0.07	-5.69	NC_022308.1	73530001	0.06	-6.45
NW_005104500.1	10001	0.07	-5.67	NC_022307.1	47630001	0.06	-6.44

NC_022319.1	30530001	0.07	-5.67	NC_022298.1	67640001	0.06	-6.42
NC_022298.1	1.13E+08	0.07	-5.66	NC_022308.1	73450001	0.06	-6.40
NC_022303.1	48840001	0.08	-5.65	NC_022294.1	96040001	0.06	-6.39
NC_022298.1	1.13E+08	0.08	-5.61	NW_005101329.1	400001	0.06	-6.38
NC_022305.1	65510001	0.08	-5.59	NC_022298.1	67670001	0.06	-6.38
NC_022314.1	56520001	0.08	-5.58	NC_022305.1	75340001	0.06	-6.36
NC_022309.1	62080001	0.08	-5.55	NW_005101032.1	1	0.06	-6.35
NC_022296.1	1.15E+08	0.08	-5.55	NC_022294.1	1.14E+08	0.06	-6.34
NW_005101191.1	1	0.08	-5.55	NC_022294.1	95970001	0.06	-6.33
NC_022319.1	30540001	0.08	-5.55	NC_022297.1	1.02E+08	0.06	-6.31
NC_022314.1	56490001	0.08	-5.54	NC_022308.1	73560001	0.06	-6.30
NC_022322.1	13590001	0.08	-5.53	NC_022297.1	22450001	0.06	-6.29
NC_022296.1	92060001	0.08	-5.52	NC_022322.1	82200001	0.06	-6.29
NC_022314.1	56510001	0.08	-5.52	NW_005101032.1	90001	0.06	-6.28
NC_022304.1	27970001	0.08	-5.51	NC_022294.1	96000001	0.06	-6.28
NC_022300.1	42030001	0.08	-5.51	NC_022297.1	22440001	0.07	-6.24
NC_022309.1	62180001	0.08	-5.51	NW_005101032.1	30001	0.07	-6.22
NC_022298.1	1.13E+08	0.08	-5.50	NW_005101032.1	40001	0.07	-6.21
NC_022299.1	23820001	0.08	-5.49	NC_022294.1	1.14E+08	0.07	-6.18
NC_022303.1	13200001	0.08	-5.49	NC_022294.1	95990001	0.07	-6.17
NC_022314.1	56500001	0.08	-5.48	NC_022308.1	73440001	0.07	-6.16
NW_005104500.1	1	0.08	-5.47	NW_005101032.1	190001	0.07	-6.15
NC_022298.1	85390001	0.08	-5.47	NC_022322.1	76110001	0.07	-6.15
NC_022303.1	13190001	0.08	-5.47	NW_005101329.1	420001	0.07	-6.15
NC_022309.1	62240001	0.08	-5.46	NW_005101032.1	170001	0.07	-6.13
NC_022300.1	42010001	0.08	-5.46	NC_022297.1	22410001	0.07	-6.12
NC_022299.1	23830001	0.09	-5.43	NC_022298.1	67660001	0.07	-6.10
NC_022298.1	1.13E+08	0.09	-5.43	NC_022298.1	67680001	0.07	-6.09
NC_022298.1	1.13E+08	0.09	-5.42	NC_022294.1	95860001	0.07	-6.07
NC_022293.1	1.43E+08	0.09	-5.41	NW_005101032.1	80001	0.07	-6.06
NC_022307.1	49380001	0.09	-5.41	NW_005101268.1	90001	0.07	-6.06
NW_005101191.1	10001	0.09	-5.40	NC_022314.1	17480001	0.07	-6.04
NW_005102490.1	80001	0.09	-5.39	NC_022305.1	75330001	0.07	-6.02
NC_022309.1	62040001	0.09	-5.39	NC_022315.1	25110001	0.08	-6.01
NC_022307.1	59990001	0.09	-5.38	NC_022315.1	25120001	0.08	-6.01
NC_022319.1	30520001	0.09	-5.38	NW_005101329.1	410001	0.08	-6.01
NC_022308.1	51360001	0.09	-5.37	NC_022293.1	73440001	0.08	-6.00
NC_022303.1	62800001	0.09	-5.37	NC_022294.1	96020001	0.08	-5.98
NC_022303.1	14960001	0.09	-5.36	NW_005101032.1	180001	0.08	-5.97
NC_022305.1	65450001	0.09	-5.36	NC_022305.1	75310001	0.08	-5.97
NC_022307.1	60000001	0.09	-5.36	NC_022293.1	64900001	0.08	-5.97
NC_022307.1	60010001	0.09	-5.36	NW_005101032.1	70001	0.08	-5.97

NW_005102490.1	70001	0.09	-5.35	NC_022294.1	95980001	0.08	-5.97
NC_022310.1	49000001	0.09	-5.35	NC_022293.1	78120001	0.08	-5.94
NC_022310.1	54450001	0.09	-5.34	NW_005101032.1	10001	0.08	-5.94
NC_022294.1	71070001	0.09	-5.33	NC_022304.1	74980001	0.08	-5.92
NC_022296.1	1.15E+08	0.09	-5.33	NW_005101032.1	60001	0.08	-5.90
NC_022297.1	63950001	0.09	-5.32	NW_005101629.1	80001	0.08	-5.90
NC_022305.1	65440001	0.09	-5.32	NC_022306.1	76280001	0.08	-5.90
NC_022317.1	510001	0.09	-5.30	NC_022297.1	1.02E+08	0.08	-5.90
NC_022305.1	65500001	0.09	-5.30	NC_022308.1	63980001	0.08	-5.88
NC_022309.1	62020001	0.09	-5.29	NC_022307.1	47600001	0.08	-5.88
NC_022322.1	13580001	0.09	-5.29	NC_022293.1	1.1E+08	0.08	-5.86
NC_022307.1	49390001	0.09	-5.27	NC_022294.1	95960001	0.08	-5.86
NC_022309.1	62060001	0.09	-5.27	NC_022311.1	20130001	0.08	-5.85
NC_022296.1	1.15E+08	0.09	-5.27	NC_022305.1	75300001	0.08	-5.84
NC_022303.1	62810001	0.09	-5.26	NC_022297.1	1.02E+08	0.08	-5.83
NC_022321.1	40740001	0.09	-5.26	NC_022305.1	75320001	0.08	-5.83
NC_022313.1	35980001	0.09	-5.25	NC_022305.1	18890001	0.08	-5.82
NC_022300.1	41990001	0.09	-5.25	NC_022307.1	47590001	0.08	-5.82
NC_022309.1	62050001	0.09	-5.25	NC_022322.1	76120001	0.08	-5.81
NC_022300.1	42020001	0.09	-5.24	NW_005101032.1	20001	0.08	-5.80
NC_022322.1	1.13E+08	0.09	-5.24	NC_022322.1	68210001	0.08	-5.80
NC_022298.1	1.13E+08	0.09	-5.23	NC_022293.1	73450001	0.08	-5.80
NC_022294.1	71060001	0.09	-5.23	NC_022308.1	63990001	0.08	-5.79
NC_022303.1	14950001	0.09	-5.23	NC_022322.1	38190001	0.08	-5.78
NC_022314.1	27990001	0.10	-5.22	NC_022305.1	18900001	0.08	-5.78
NC_022309.1	62090001	0.10	-5.22	NC_022293.1	73430001	0.09	-5.77
NC_022309.1	62100001	0.10	-5.22	NC_022322.1	82680001	0.09	-5.77
NC_022293.1	1.19E+08	0.10	-5.22	NC_022298.1	67610001	0.09	-5.77
NC_022298.1	1.13E+08	0.10	-5.22	NW_005102490.1	180001	0.09	-5.76
NC_022298.1	10700001	0.10	-5.21	NC_022298.1	68430001	0.09	-5.76
NC_022309.1	62120001	0.10	-5.20	NC_022308.1	64000001	0.09	-5.75
NW_005101191.1	20001	0.10	-5.20	NC_022293.1	78100001	0.09	-5.74
NC_022305.1	65480001	0.10	-5.18	NC_022293.1	64910001	0.09	-5.72
NC_022305.1	65460001	0.10	-5.17	NC_022294.1	1.14E+08	0.09	-5.71
NC_022303.1	14980001	0.10	-5.15	NC_022293.1	73460001	0.09	-5.70
NC_022303.1	13180001	0.10	-5.15	NC_022297.1	1.02E+08	0.09	-5.70
NC_022309.1	62230001	0.10	-5.14	NC_022321.1	46430001	0.09	-5.68
NC_022322.1	23490001	0.10	-5.14	NC_022298.1	67690001	0.09	-5.66
NC_022318.1	23820001	0.10	-5.13	NC_022300.1	300001	0.09	-5.66
NC_022303.1	48770001	0.10	-5.10	NC_022322.1	38220001	0.09	-5.66
NC_022309.1	62030001	0.10	-5.10	NC_022322.1	68200001	0.09	-5.66
NC_022300.1	41860001	0.10	-5.09	NC_022293.1	73400001	0.09	-5.65

NC_022305.1	31410001	0.10	-5.09	NC_022293.1	73420001	0.09	-5.64
NC_022309.1	34170001	0.10	-5.08	NC_022322.1	38200001	0.09	-5.64
NC_022304.1	33390001	0.10	-5.08	NW_005101329.1	390001	0.09	-5.64
NC_022305.1	31400001	0.10	-5.08	NW_005101329.1	430001	0.09	-5.64
NC_022304.1	33420001	0.10	-5.07	NC_022322.1	38210001	0.09	-5.64
NC_022322.1	1.14E+08	0.10	-5.06	NW_005101329.1	440001	0.09	-5.63
NC_022300.1	41980001	0.10	-5.06	NC_022293.1	64890001	0.09	-5.63
NC_022303.1	14970001	0.10	-5.06	NW_005101629.1	90001	0.09	-5.62
NC_022307.1	59980001	0.10	-5.06	NC_022314.1	17470001	0.09	-5.60
NC_022309.1	62190001	0.10	-5.05	NC_022313.1	9510001	0.09	-5.59
NC_022307.1	60020001	0.10	-5.03	NC_022322.1	82210001	0.09	-5.58
NC_022304.1	33410001	0.10	-5.03	NC_022313.1	9520001	0.09	-5.58
NC_022313.1	35990001	0.10	-5.03	NC_022293.1	73410001	0.09	-5.58
NC_022298.1	68660001	0.10	-5.02	NC_022312.1	31530001	0.09	-5.57
NC_022322.1	1.08E+08	0.10	-5.02	NW_005101032.1	120001	0.10	-5.54
NC_022310.1	49010001	0.10	-5.01	NC_022302.1	49580001	0.10	-5.54
NC_022294.1	16780001	0.10	-5.01	NC_022300.1	290001	0.10	-5.54
NC_022322.1	42310001	0.10	-5.01	NC_022304.1	74990001	0.10	-5.53
NC_022308.1	51350001	0.10	-5.01	NC_022300.1	42650001	0.10	-5.53
NC_022318.1	23810001	0.11	-5.00	NC_022302.1	49160001	0.10	-5.52
				NC_022300.1	40740001	0.10	-5.51
				NC_022312.1	31520001	0.10	-5.51
				NW_005101032.1	100001	0.10	-5.51
				NC_022322.1	82690001	0.10	-5.51
				NW_005102164.1	70001	0.10	-5.51
				NC_022308.1	64010001	0.10	-5.49
				NC_022322.1	82670001	0.10	-5.49
				NC_022322.1	68220001	0.10	-5.48
				NC_022310.1	34250001	0.10	-5.47
				NC_022311.1	20140001	0.10	-5.45
				NC_022314.1	17490001	0.10	-5.45
				NC_022315.1	9790001	0.10	-5.44
				NC_022322.1	68230001	0.10	-5.44
				NC_022311.1	20030001	0.10	-5.44
				NC_022293.1	64880001	0.10	-5.43
				NW_005101032.1	110001	0.10	-5.43

Supplementary table 3 list of candidates of genetic differentiation

CHROM	POS	Fst	ZFst>4.5
NC_022322.1	26320001	0.80	7.50
NC_022322.1	26330001	0.77	7.24
NC_022322.1	26310001	0.75	7.02

NC_022322.1	24620001	0.74	6.83
NC_022294.1	1.28E+08	0.72	6.66
NC_022299.1	72630001	0.72	6.65
NC_022322.1	24630001	0.72	6.65
NC_022305.1	75340001	0.72	6.62
NC_022322.1	24610001	0.71	6.58
NC_022294.1	1.28E+08	0.71	6.49
NC_022299.1	72620001	0.70	6.48
NC_022294.1	1.28E+08	0.69	6.36
NC_022299.1	72640001	0.69	6.35
NC_022305.1	75330001	0.69	6.34
NC_022322.1	26300001	0.69	6.29
NC_022322.1	24640001	0.69	6.29
NC_022294.1	1.28E+08	0.68	6.25
NC_022322.1	68130001	0.67	6.10
NC_022311.1	26330001	0.67	6.09
NC_022322.1	42200001	0.67	6.08
NC_022305.1	45230001	0.67	6.07
NC_022322.1	26340001	0.67	6.05
NC_022322.1	24650001	0.66	6.04
NC_022305.1	75320001	0.66	6.01
NC_022305.1	75350001	0.66	5.99
NC_022322.1	13250001	0.66	5.97
NW_005114481.1	1	0.66	5.97
NC_022294.1	1.28E+08	0.66	5.96
NC_022305.1	75310001	0.66	5.95
NC_022305.1	49440001	0.66	5.93
NC_022314.1	21790001	0.65	5.92
NC_022305.1	65220001	0.65	5.92
NC_022305.1	49450001	0.65	5.90
NC_022305.1	45240001	0.65	5.88
NC_022322.1	42210001	0.65	5.88
NC_022314.1	21800001	0.65	5.87
NC_022322.1	42190001	0.65	5.87
NC_022300.1	36820001	0.65	5.87
NC_022300.1	36830001	0.65	5.86
NC_022322.1	24600001	0.65	5.86
NC_022301.1	79710001	0.65	5.83
NC_022322.1	42220001	0.64	5.81
NC_022301.1	79700001	0.64	5.81
NC_022311.1	26320001	0.64	5.81

NC_022322.1	13230001	0.64	5.81
NC_022314.1	21830001	0.64	5.80
NC_022322.1	26290001	0.64	5.80
NC_022322.1	68120001	0.64	5.78
NC_022301.1	79720001	0.64	5.77
NC_022314.1	21810001	0.64	5.75
NC_022322.1	68070001	0.64	5.75
NC_022305.1	65230001	0.64	5.75
NC_022305.1	49460001	0.64	5.75
NC_022322.1	13260001	0.64	5.73
NC_022322.1	13240001	0.64	5.73
NC_022314.1	21820001	0.64	5.73
NC_022322.1	68090001	0.64	5.73
NC_022322.1	42230001	0.64	5.71
NC_022302.1	49740001	0.64	5.71
NC_022300.1	38530001	0.64	5.71
NC_022299.1	72650001	0.63	5.70
NC_005044.2	1	0.63	5.69
NC_022322.1	68110001	0.63	5.68
NC_022302.1	49730001	0.63	5.68
NC_022314.1	21860001	0.63	5.66
NC_022322.1	68100001	0.63	5.66
NC_022300.1	36840001	0.63	5.66
NC_022300.1	38540001	0.63	5.65
NC_022300.1	38520001	0.63	5.64
NC_022305.1	65240001	0.63	5.63
NC_022300.1	35960001	0.63	5.62
NC_022314.1	21850001	0.62	5.60
NC_022305.1	45250001	0.62	5.57
NC_022302.1	49720001	0.62	5.57
NC_022311.1	26340001	0.62	5.56
NC_022305.1	62940001	0.62	5.56
NC_022322.1	68140001	0.62	5.56
NC_022300.1	36850001	0.62	5.55
NC_022305.1	75300001	0.62	5.55
NC_022305.1	49470001	0.62	5.55
NC_022299.1	56650001	0.62	5.55
NC_022305.1	49610001	0.62	5.53
NC_005044.2	10001	0.62	5.51
NC_022322.1	42240001	0.62	5.50
NC_022305.1	62930001	0.62	5.50

NC_022303.1	47740001	0.62	5.49
NC_022322.1	67880001	0.61	5.49
NC_022316.1	25680001	0.61	5.49
NC_022302.1	49760001	0.61	5.47
NC_022302.1	45440001	0.61	5.46
NC_022296.1	18220001	0.61	5.46
NC_022314.1	27990001	0.61	5.46
NC_022316.1	25690001	0.61	5.46
NC_022301.1	79690001	0.61	5.45
NC_022299.1	46850001	0.61	5.45
NC_022318.1	19270001	0.61	5.45
NC_022300.1	35950001	0.61	5.45
NC_022298.1	92210001	0.61	5.45
NC_022299.1	46830001	0.61	5.44
NC_022303.1	47750001	0.61	5.43
NC_022314.1	21840001	0.61	5.43
NC_022322.1	68150001	0.61	5.42
NC_022322.1	68060001	0.61	5.42
NC_022304.1	70640001	0.61	5.41
NC_022302.1	49750001	0.61	5.41
NC_022322.1	68080001	0.61	5.41
NC_022299.1	56660001	0.61	5.41
NC_022304.1	70630001	0.61	5.41
NC_022322.1	42180001	0.61	5.41
NC_022300.1	38550001	0.61	5.40
NC_022299.1	15550001	0.61	5.40
NC_022322.1	67890001	0.61	5.40
NC_022305.1	49430001	0.61	5.39
NC_022303.1	47800001	0.61	5.38
NC_022322.1	24590001	0.61	5.38
NC_022314.1	21780001	0.60	5.38
NC_022322.1	26350001	0.60	5.38
NC_022300.1	36860001	0.60	5.37
NC_022297.1	22880001	0.60	5.36
NC_022306.1	73890001	0.60	5.36
NC_022301.1	79730001	0.60	5.34
NC_022305.1	62920001	0.60	5.32
NC_022311.1	26310001	0.60	5.32
NC_022299.1	46840001	0.60	5.32
NC_022305.1	65210001	0.60	5.30
NC_022294.1	1.28E+08	0.60	5.29

NC_022300.1	35970001	0.60	5.29
NC_022316.1	25670001	0.60	5.29
NC_022305.1	64090001	0.60	5.28
NC_022305.1	49600001	0.60	5.27
NC_022322.1	67870001	0.60	5.27
NC_022302.1	50610001	0.60	5.27
NC_022298.1	92190001	0.59	5.27
NC_022305.1	46360001	0.59	5.26
NC_022322.1	67760001	0.59	5.26
NC_022303.1	47730001	0.59	5.25
NC_022306.1	73880001	0.59	5.25
NC_022305.1	64080001	0.59	5.25
NC_022299.1	72610001	0.59	5.24
NC_022322.1	42250001	0.59	5.24
NC_022322.1	8640001	0.59	5.23
NC_022322.1	67850001	0.59	5.23
NC_022305.1	46370001	0.59	5.23
NC_022305.1	65200001	0.59	5.22
NC_022300.1	37080001	0.59	5.22
NC_022303.1	47790001	0.59	5.22
NC_022302.1	49780001	0.59	5.21
NC_022302.1	49710001	0.59	5.20
NC_022306.1	73900001	0.59	5.20
NC_022299.1	15540001	0.59	5.18
NC_022322.1	42260001	0.59	5.17
NC_022298.1	92200001	0.59	5.16
NC_022305.1	62910001	0.59	5.16
NC_022322.1	42170001	0.59	5.16
NC_022299.1	56640001	0.58	5.15
NC_022322.1	68160001	0.58	5.15
NC_022302.1	49770001	0.58	5.15
NC_022305.1	64100001	0.58	5.15
NC_022317.1	9650001	0.58	5.14
NC_022302.1	45450001	0.58	5.13
NC_022316.1	25700001	0.58	5.13
NC_022303.1	47810001	0.58	5.12
NC_022303.1	47830001	0.58	5.12
NC_022322.1	67860001	0.58	5.12
NC_022300.1	1270001	0.58	5.12
NC_022302.1	47850001	0.58	5.11
NC_022322.1	24660001	0.58	5.11

NC_022296.1	92060001	0.58	5.11
NC_022300.1	37090001	0.58	5.11
NC_022303.1	47760001	0.58	5.10
NC_022299.1	56630001	0.58	5.10
NC_022302.1	84900001	0.58	5.10
NC_022302.1	84910001	0.58	5.09
NC_022300.1	37070001	0.58	5.09
NC_022319.1	18620001	0.58	5.08
NC_022322.1	68050001	0.58	5.08
NC_022322.1	8650001	0.58	5.06
NC_022302.1	50600001	0.58	5.06
NC_022303.1	47820001	0.58	5.06
NC_022312.1	69220001	0.58	5.06
NC_022304.1	70650001	0.58	5.05
NC_022296.1	92050001	0.58	5.05
NC_022300.1	38510001	0.58	5.05
NC_022305.1	65250001	0.58	5.05
NC_022314.1	56530001	0.57	5.05
NC_022302.1	45430001	0.57	5.04
NC_022322.1	42100001	0.57	5.02
NC_022296.1	92070001	0.57	5.01
NC_022297.1	22870001	0.57	5.01
NC_022299.1	46820001	0.57	5.01
NC_022302.1	84920001	0.57	5.00
NC_022310.1	34630001	0.57	5.00
NC_022307.1	45530001	0.57	5.00
NC_022317.1	9660001	0.57	4.99
NC_022316.1	25660001	0.57	4.99
NC_022306.1	30130001	0.57	4.99
NC_022302.1	52040001	0.57	4.99
NC_022302.1	84890001	0.57	4.98
NC_022302.1	45460001	0.57	4.98
NC_022305.1	75360001	0.57	4.98
NC_022316.1	25720001	0.57	4.98
NC_022314.1	21770001	0.57	4.96
NC_022302.1	47860001	0.57	4.96
NC_022300.1	38560001	0.57	4.96
NC_022312.1	69210001	0.57	4.96
NC_022322.1	13220001	0.57	4.96
NC_022305.1	45260001	0.57	4.96
NC_022294.1	26060001	0.57	4.95

NC_022296.1	18210001	0.57	4.95
NC_022322.1	67840001	0.57	4.95
NC_022322.1	42160001	0.57	4.95
NC_022318.1	19280001	0.57	4.94
NC_022296.1	18230001	0.57	4.94
NC_022296.1	18240001	0.56	4.93
NC_022305.1	64070001	0.56	4.93
NC_022318.1	19260001	0.56	4.93
NC_022296.1	92040001	0.56	4.93
NC_022305.1	49590001	0.56	4.93
NC_022319.1	18630001	0.56	4.92
NC_022298.1	92220001	0.56	4.92
NC_022322.1	67830001	0.56	4.92
NC_022314.1	4930001	0.56	4.91
NC_022294.1	1.28E+08	0.56	4.91
NC_022322.1	67910001	0.56	4.90
NC_022322.1	67770001	0.56	4.90
NC_022296.1	96880001	0.56	4.90
NC_022305.1	65510001	0.56	4.89
NC_022310.1	33850001	0.56	4.89
NC_022310.1	34650001	0.56	4.89
NC_022302.1	84880001	0.56	4.88
NC_022322.1	42130001	0.56	4.88
NC_022294.1	26070001	0.56	4.88
NC_022296.1	92030001	0.56	4.87
NC_022313.1	3810001	0.56	4.87
NC_022314.1	21870001	0.56	4.87
NC_022322.1	42120001	0.56	4.86
NC_022316.1	25710001	0.56	4.86
NW_005106983.1	10001	0.56	4.86
NC_022299.1	46860001	0.56	4.85
NC_022306.1	30140001	0.56	4.85
NC_022303.1	47770001	0.56	4.84
NC_022299.1	56670001	0.56	4.84
NC_022306.1	30120001	0.56	4.84
NC_022305.1	65190001	0.56	4.84
NC_022305.1	27000001	0.56	4.83
NC_022306.1	73910001	0.55	4.82
NC_022302.1	49800001	0.55	4.82
NC_022302.1	50620001	0.55	4.82
NC_022305.1	65520001	0.55	4.81

NC_022311.1	26350001	0.55	4.80
NC_022322.1	13270001	0.55	4.80
NC_022300.1	1280001	0.55	4.80
NC_022305.1	46350001	0.55	4.80
NW_005104722.1	1	0.55	4.80
NC_022322.1	67900001	0.55	4.79
NC_022305.1	65180001	0.55	4.79
NC_022314.1	56500001	0.55	4.79
NC_022300.1	38600001	0.55	4.78
NC_022295.1	90460001	0.55	4.78
NC_022298.1	92180001	0.55	4.78
NC_022305.1	45220001	0.55	4.78
NC_022322.1	42270001	0.55	4.77
NC_022322.1	42010001	0.55	4.77
NC_022305.1	46380001	0.55	4.77
NC_022305.1	65500001	0.55	4.76
NC_022310.1	34640001	0.55	4.76
NC_022305.1	63970001	0.55	4.76
NC_022305.1	49580001	0.55	4.76
NC_022297.1	22890001	0.55	4.76
NC_022305.1	62950001	0.55	4.75
NC_022299.1	15560001	0.55	4.74
NC_022300.1	38620001	0.55	4.74
NC_022322.1	42140001	0.55	4.74
NC_022322.1	68190001	0.55	4.74
NC_022322.1	42110001	0.55	4.73
NW_005112027.1	1	0.55	4.73
NC_022302.1	49810001	0.55	4.73
NC_022305.1	64110001	0.55	4.73
NC_022305.1	26990001	0.55	4.73
NC_022302.1	50630001	0.55	4.72
NC_022322.1	67800001	0.55	4.72
NC_022322.1	42000001	0.55	4.72
NC_022306.1	73920001	0.54	4.71
NC_022307.1	45540001	0.54	4.71
NC_022310.1	34660001	0.54	4.71
NC_022322.1	24580001	0.54	4.71
NC_022302.1	49790001	0.54	4.71
NC_022322.1	67820001	0.54	4.71
NC_022299.1	15530001	0.54	4.70
NC_022305.1	49480001	0.54	4.70

NC_022300.1	35980001	0.54	4.69
NC_022302.1	49700001	0.54	4.69
NC_022302.1	50590001	0.54	4.69
NC_022310.1	33820001	0.54	4.69
NC_022319.1	18640001	0.54	4.69
NC_022317.1	40290001	0.54	4.69
NC_022300.1	37100001	0.54	4.69
NC_022295.1	90440001	0.54	4.68
NC_022322.1	68040001	0.54	4.68
NC_022305.1	62960001	0.54	4.68
NC_022298.1	10700001	0.54	4.68
NC_022314.1	28000001	0.54	4.68
NC_022322.1	68170001	0.54	4.68
NC_022303.1	47780001	0.54	4.68
NC_022314.1	4940001	0.54	4.67
NC_022312.1	69230001	0.54	4.66
NW_005115693.1	1	0.54	4.66
NC_022322.1	68200001	0.54	4.66
NC_022305.1	63950001	0.54	4.65
NC_022307.1	45520001	0.54	4.65
NC_022305.1	63910001	0.54	4.64
NC_022322.1	2550001	0.54	4.64
NC_022314.1	26590001	0.54	4.64
NC_022296.1	92020001	0.54	4.63
NC_022315.1	15590001	0.54	4.63
NC_022300.1	38590001	0.54	4.62
NC_022302.1	52030001	0.54	4.62
NC_022316.1	25730001	0.54	4.62
NC_022305.1	63940001	0.54	4.61
NC_022298.1	96970001	0.54	4.61
NC_022322.1	42150001	0.53	4.60
NC_022304.1	70620001	0.53	4.60
NC_022294.1	1.08E+08	0.53	4.59
NC_022317.1	9640001	0.53	4.59
NC_022322.1	8630001	0.53	4.59
NC_022301.1	79740001	0.53	4.59
NC_022305.1	65160001	0.53	4.58
NC_022322.1	41990001	0.53	4.58
NC_022310.1	34620001	0.53	4.58
NC_022300.1	36810001	0.53	4.58
NC_022300.1	38610001	0.53	4.58

NC_022295.1	64130001	0.53	4.58
NC_022322.1	67810001	0.53	4.57
NC_022300.1	36730001	0.53	4.57
NC_022302.1	84930001	0.53	4.57
NC_022316.1	25750001	0.53	4.57
NC_022305.1	63930001	0.53	4.57
NC_022322.1	38580001	0.53	4.56
NC_022322.1	67920001	0.53	4.56
NC_022322.1	68210001	0.53	4.56
NC_022300.1	35940001	0.53	4.56
NC_022300.1	36740001	0.53	4.56
NC_022302.1	84940001	0.53	4.56
NC_022314.1	56490001	0.53	4.56
NC_022306.1	73930001	0.53	4.55
NC_022295.1	90450001	0.53	4.55
NW_005103886.1	1	0.53	4.54
NC_022301.1	26710001	0.53	4.54
NC_022322.1	91860001	0.53	4.54
NC_022298.1	10710001	0.53	4.54
NC_022296.1	96870001	0.53	4.54
NC_022300.1	1260001	0.53	4.53
NC_022302.1	39760001	0.53	4.53
NC_022298.1	96960001	0.53	4.53
NW_005101059.1	1200001	0.53	4.53
NC_022310.1	21220001	0.53	4.53
NC_022322.1	68000001	0.53	4.52
NC_022300.1	38630001	0.53	4.52
NC_022305.1	26980001	0.53	4.52
NC_022319.1	18610001	0.53	4.52
NC_022305.1	64060001	0.53	4.52
NC_022313.1	3800001	0.53	4.52
NW_005104910.1	1	0.53	4.51
NC_022315.1	42370001	0.53	4.51
NC_022296.1	96890001	0.53	4.51
NC_022300.1	38640001	0.53	4.51
NC_022314.1	4920001	0.53	4.50
NC_022322.1	42280001	0.53	4.50

Supplementary table 4 Regions that contained both low heterozygosity and high genetic differentiation

DBG	IMCG

CHROM	POS	Fst	ZFst	HP	ZHP	ZFst+|ZHP|	CHROM	POS	Fst	ZFst	HP	ZHP	ZFst+|ZHP|
NC_022305.1	75340001	0.72	6.35	0.06	-6.36	12.71	NC_022314.1	56530001	0.57	4.86	0.03	-6.55	11.40
NC_022305.1	75330001	0.69	6.08	0.07	-6.02	12.10	NC_022300.1	41890001	0.51	4.18	0.01	-7.17	11.35
NC_022305.1	75310001	0.66	5.72	0.08	-5.97	11.69	NC_022296.1	92030001	0.56	4.69	0.04	-6.35	11.04
NC_022305.1	75320001	0.66	5.78	0.08	-5.83	11.61	NC_022296.1	92040001	0.56	4.75	0.05	-6.29	11.04
NC_022305.1	75300001	0.62	5.33	0.08	-5.84	11.17	NC_022296.1	92050001	0.58	4.86	0.06	-6.04	10.90
NC_022322.1	68130001	0.67	5.85	0.11	-5.27	11.13	NC_022300.1	41880001	0.52	4.26	0.04	-6.53	10.79
NC_022305.1	49450001	0.65	5.66	0.10	-5.33	11.00	NC_022314.1	27990001	0.61	5.25	0.10	-5.22	10.47
NC_022305.1	75350001	0.66	5.75	0.11	-5.17	10.92	NC_022296.1	92060001	0.58	4.92	0.08	-5.52	10.44
NC_022322.1	68070001	0.64	5.53	0.11	-5.22	10.75	NC_022322.1	26320001	0.80	7.19	0.19	-3.24	10.43
NC_022304.1	70640001	0.61	5.20	0.10	-5.36	10.56	NC_022305.1	65520001	0.55	4.63	0.07	-5.75	10.38
NC_022305.1	49460001	0.64	5.52	0.12	-4.97	10.49	NC_022300.1	41900001	0.45	3.52	0.02	-6.84	10.35
NC_022294.1	1.28E+08	0.72	6.39	0.16	-4.09	10.48	NC_022311.1	26330001	0.67	5.85	0.13	-4.48	10.32
NC_022305.1	49440001	0.66	5.70	0.13	-4.77	10.47	NC_022305.1	65510001	0.56	4.71	0.08	-5.59	10.30
NC_022297.1	22430001	0.49	3.92	0.06	-6.45	10.37	NC_022322.1	42230001	0.64	5.49	0.12	-4.74	10.23
NC_022297.1	22420001	0.48	3.86	0.05	-6.50	10.36	NC_022305.1	65530001	0.52	4.26	0.06	-5.89	10.15
NC_022294.1	1.28E+08	0.71	6.23	0.16	-4.09	10.32	NC_022300.1	41870001	0.51	4.21	0.06	-5.92	10.14
NC_022322.1	68210001	0.53	4.39	0.08	-5.80	10.19	NC_022314.1	56500001	0.55	4.61	0.08	-5.48	10.09
NC_022294.1	1.28E+08	0.68	6.00	0.15	-4.18	10.18	NC_022311.1	26320001	0.64	5.58	0.13	-4.44	10.02
NC_022304.1	70650001	0.58	4.86	0.10	-5.31	10.17	NC_022322.1	24620001	0.74	6.55	0.18	-3.47	10.02
NC_022322.1	68200001	0.54	4.49	0.09	-5.66	10.15	NC_022322.1	24610001	0.71	6.31	0.17	-3.66	9.97
NC_022297.1	22410001	0.48	3.88	0.07	-6.12	10.00	NC_022322.1	42210001	0.65	5.65	0.14	-4.31	9.96
NC_022322.1	68090001	0.64	5.50	0.14	-4.49	9.99	NC_022293.1	1.43E+08	0.45	3.58	0.04	-6.37	9.95
NC_022300.1	36820001	0.65	5.64	0.15	-4.32	9.95	NC_022305.1	65540001	0.51	4.15	0.07	-5.80	9.94
NC_022322.1	68120001	0.64	5.55	0.15	-4.32	9.87	NC_022314.1	56490001	0.53	4.39	0.08	-5.54	9.93
NC_022305.1	75360001	0.57	4.79	0.12	-5.06	9.85	NC_022305.1	65500001	0.55	4.59	0.09	-5.30	9.88
NC_022305.1	45230001	0.67	5.83	0.16	-3.95	9.77	NC_022314.1	56520001	0.52	4.25	0.08	-5.58	9.83
NC_022322.1	68110001	0.63	5.46	0.15	-4.29	9.76	NC_022322.1	42240001	0.62	5.29	0.13	-4.53	9.82
NC_022314.1	21790001	0.65	5.69	0.16	-4.05	9.74	NC_022314.1	56510001	0.52	4.29	0.08	-5.52	9.81
NC_022298.1	68430001	0.49	3.98	0.09	-5.76	9.73	NC_022322.1	42200001	0.67	5.84	0.15	-3.96	9.81
NC_022322.1	68190001	0.55	4.56	0.11	-5.15	9.71	NC_022322.1	42220001	0.64	5.58	0.14	-4.22	9.80
NC_022322.1	68060001	0.61	5.21	0.14	-4.49	9.70	NC_022298.1	10700001	0.54	4.51	0.10	-5.21	9.72
NC_022297.1	22450001	0.43	3.38	0.06	-6.29	9.68	NC_022309.1	62150001	0.41	3.15	0.03	-6.54	9.69
NC_022322.1	68230001	0.51	4.19	0.10	-5.44	9.63	NC_022296.1	92070001	0.57	4.82	0.11	-4.85	9.67
NC_022322.1	68010001	0.52	4.30	0.10	-5.32	9.63	NC_022309.1	62130001	0.43	3.35	0.04	-6.32	9.67
NC_022322.1	68100001	0.63	5.44	0.15	-4.18	9.62	NC_022309.1	62240001	0.51	4.20	0.08	-5.46	9.66
NC_022322.1	68220001	0.51	4.14	0.10	-5.48	9.62	NC_022309.1	62140001	0.43	3.34	0.05	-6.31	9.65

NC_022297.1	22440001	0.43	3.37	0.07	-6.24	9.61	NC_022309.1	62020001	0.52	4.29	0.09	-5.29	9.58
NC_022300.1	36830001	0.65	5.63	0.16	-3.95	9.58	NC_022309.1	62160001	0.40	3.05	0.04	-6.45	9.49
NC_022310.1	34250001	0.50	4.09	0.10	-5.47	9.56	NC_022305.1	65480001	0.52	4.28	0.10	-5.18	9.46
NW_005101629.1	80001	0.46	3.66	0.08	-5.90	9.55	NC_022322.1	42250001	0.59	5.04	0.13	-4.36	9.40
NC_022322.1	67880001	0.61	5.28	0.15	-4.22	9.49	NC_022305.1	62940001	0.62	5.34	0.15	-4.03	9.37
NC_022294.1	1.28E+08	0.60	5.09	0.15	-4.36	9.45	NC_022300.1	41910001	0.40	3.02	0.05	-6.31	9.33
NC_022322.1	67850001	0.59	5.03	0.14	-4.41	9.44	NC_022322.1	13250001	0.66	5.73	0.17	-3.56	9.29
NC_022322.1	68140001	0.62	5.34	0.16	-4.07	9.42	NC_022311.1	26310001	0.60	5.12	0.14	-4.15	9.27
NW_005101629.1	90001	0.47	3.77	0.09	-5.62	9.39	NC_022305.1	65460001	0.50	4.10	0.10	-5.17	9.27
NC_022322.1	68000001	0.53	4.36	0.12	-5.00	9.36	NC_022305.1	65450001	0.48	3.89	0.09	-5.36	9.25
NC_022322.1	67870001	0.60	5.07	0.15	-4.28	9.35	NC_022309.1	62170001	0.41	3.15	0.06	-6.08	9.23
NC_022322.1	68050001	0.58	4.89	0.14	-4.46	9.34	NC_022305.1	63930001	0.53	4.40	0.11	-4.82	9.22
NC_022308.1	73560001	0.40	3.03	0.06	-6.30	9.33	NC_022305.1	62960001	0.54	4.51	0.12	-4.70	9.21
NC_022305.1	49470001	0.62	5.33	0.16	-3.98	9.32	NC_022314.1	27980001	0.52	4.29	0.11	-4.89	9.18
NC_022322.1	67860001	0.58	4.92	0.15	-4.32	9.24	NC_022311.1	26340001	0.62	5.34	0.16	-3.81	9.16
NC_022300.1	96330001	0.48	3.83	0.10	-5.40	9.24	NC_022305.1	65160001	0.53	4.42	0.12	-4.74	9.15
NC_022304.1	70660001	0.51	4.16	0.11	-5.08	9.24	NC_022305.1	65440001	0.48	3.83	0.09	-5.32	9.15
NC_022314.1	21860001	0.63	5.44	0.17	-3.72	9.17	NC_022305.1	63950001	0.54	4.48	0.12	-4.65	9.13
NC_022322.1	67990001	0.51	4.20	0.12	-4.96	9.16	NC_022310.1	49000001	0.47	3.78	0.09	-5.35	9.12
NC_022304.1	70630001	0.61	5.20	0.16	-3.95	9.15	NC_022305.1	63970001	0.55	4.58	0.13	-4.51	9.09
NC_022317.1	40280001	0.52	4.25	0.12	-4.89	9.14	NC_022322.1	24600001	0.65	5.63	0.18	-3.44	9.07
NC_022317.1	40270001	0.50	4.09	0.12	-5.03	9.12	NC_022322.1	42310001	0.50	4.05	0.10	-5.01	9.06
NC_022322.1	68080001	0.61	5.20	0.16	-3.91	9.11	NC_022305.1	65490001	0.52	4.24	0.11	-4.81	9.05
NC_022298.1	92140001	0.50	4.11	0.12	-4.98	9.10	NC_022305.1	63940001	0.54	4.44	0.12	-4.59	9.03
NC_022305.1	75370001	0.49	3.99	0.11	-5.10	9.09	NC_022314.1	28000001	0.54	4.50	0.13	-4.50	9.01
NC_022317.1	40290001	0.54	4.52	0.14	-4.53	9.05	NC_022299.1	56660001	0.61	5.20	0.16	-3.79	8.99
NC_022322.1	67760001	0.59	5.06	0.16	-3.95	9.01	NC_022306.1	73890001	0.60	5.15	0.16	-3.83	8.98
NC_022317.1	40260001	0.49	3.96	0.12	-5.05	9.00	NC_022305.1	63960001	0.52	4.23	0.12	-4.74	8.97
NC_022322.1	68150001	0.61	5.21	0.17	-3.77	8.98	NC_022322.1	42260001	0.59	4.97	0.15	-3.96	8.93
NC_022305.1	45240001	0.65	5.65	0.19	-3.31	8.96	NC_022322.1	13450001	0.42	3.19	0.07	-5.73	8.92
NC_022322.1	67980001	0.51	4.21	0.13	-4.75	8.96	NC_022305.1	65360001	0.51	4.16	0.12	-4.76	8.92
NC_022298.1	92150001	0.47	3.81	0.12	-5.06	8.87	NC_022305.1	62930001	0.62	5.28	0.17	-3.62	8.91
NC_022322.1	67890001	0.61	5.19	0.18	-3.65	8.84	NC_022313.1	3800001	0.53	4.35	0.13	-4.55	8.90
NC_022322.1	58310001	0.49	3.99	0.12	-4.84	8.83	NC_022305.1	65470001	0.49	3.94	0.11	-4.94	8.88
NC_022322.1	67840001	0.57	4.76	0.16	-4.06	8.82	NC_022296.1	92020001	0.54	4.46	0.13	-4.42	8.88
NC_022305.1	14690001	0.46	3.61	0.11	-5.18	8.79	NC_022305.1	63920001	0.51	4.16	0.12	-4.72	8.88
NC_022300.1	96340001	0.46	3.69	0.11	-5.11	8.79	NC_022309.1	62010001	0.52	4.31	0.13	-4.53	8.84
NC_022314.1	21800001	0.65	5.64	0.20	-3.14	8.78	NC_022322.1	42290001	0.51	4.22	0.12	-4.61	8.83

NC_022322.1	68020001	0.51	4.18	0.14	-4.60	8.78	NC_022314.1	56480001	0.48	3.88	0.11	-4.93	8.80
NC_022310.1	34260001	0.47	3.78	0.12	-4.97	8.75	NC_022322.1	13590001	0.42	3.27	0.08	-5.53	8.80
NC_022317.1	40250001	0.45	3.54	0.11	-5.21	8.75	NC_022309.1	62230001	0.46	3.65	0.10	-5.14	8.79
NC_022322.1	68040001	0.54	4.51	0.15	-4.22	8.73	NC_022305.1	63990001	0.52	4.25	0.13	-4.53	8.78
NC_022300.1	35970001	0.60	5.09	0.18	-3.63	8.72	NC_022311.1	26350001	0.55	4.62	0.14	-4.15	8.78
NC_022314.1	21780001	0.60	5.17	0.18	-3.48	8.64	NC_022305.1	63910001	0.54	4.47	0.14	-4.30	8.77
NC_022314.1	21870001	0.56	4.69	0.16	-3.95	8.64	NC_022308.1	51360001	0.44	3.40	0.09	-5.37	8.77
NC_022322.1	58300001	0.49	3.96	0.13	-4.66	8.62	NC_022322.1	23500001	0.48	3.88	0.11	-4.85	8.74
NC_022322.1	67830001	0.56	4.73	0.17	-3.88	8.61	NC_022298.1	96960001	0.53	4.37	0.13	-4.35	8.72
NC_022322.1	68160001	0.58	4.96	0.18	-3.64	8.60	NC_022322.1	13260001	0.64	5.51	0.19	-3.20	8.71
NW_005101629.1	60001	0.43	3.36	0.11	-5.24	8.60	NC_022303.1	13180001	0.45	3.55	0.10	-5.15	8.70
NC_022293.1	69830001	0.43	3.35	0.11	-5.21	8.56	NC_022298.1	96950001	0.50	4.09	0.12	-4.60	8.70
NC_022305.1	49430001	0.61	5.18	0.19	-3.37	8.55	NC_022299.1	56650001	0.62	5.33	0.18	-3.35	8.69
NC_022322.1	58320001	0.49	3.96	0.14	-4.59	8.55	NC_022298.1	96970001	0.54	4.44	0.14	-4.23	8.67
NC_022322.1	68030001	0.53	4.34	0.15	-4.18	8.52	NC_022322.1	23490001	0.45	3.52	0.10	-5.14	8.66
NC_022310.1	34240001	0.44	3.49	0.12	-5.02	8.51	NC_022305.1	62950001	0.55	4.57	0.15	-4.08	8.66
NC_022305.1	45220001	0.55	4.60	0.16	-3.91	8.51	NC_022305.1	45250001	0.62	5.36	0.18	-3.27	8.63
NC_022294.1	1.28E+08	0.56	4.72	0.17	-3.78	8.50	NC_022322.1	42300001	0.50	4.08	0.13	-4.55	8.63
NC_022322.1	68180001	0.52	4.30	0.15	-4.18	8.49	NC_022309.1	62030001	0.45	3.52	0.10	-5.10	8.62
NC_022306.1	30120001	0.56	4.66	0.17	-3.79	8.45	NC_022299.1	46830001	0.61	5.23	0.18	-3.39	8.62
NC_022293.1	83550001	0.44	3.47	0.12	-4.98	8.44	NC_022310.1	49010001	0.46	3.60	0.10	-5.01	8.62
NC_022293.1	69820001	0.42	3.20	0.11	-5.15	8.36	NC_022322.1	23510001	0.49	3.92	0.12	-4.70	8.61
NC_022322.1	58580001	0.43	3.36	0.12	-4.98	8.33	NC_022306.1	73900001	0.59	5.00	0.17	-3.61	8.61
NW_005101629.1	70001	0.41	3.13	0.11	-5.18	8.31	NC_022299.1	46850001	0.61	5.24	0.18	-3.37	8.61
NC_022302.1	52040001	0.57	4.80	0.18	-3.48	8.28	NW_005101047.1	560001	0.46	3.66	0.11	-4.95	8.60
NC_022295.1	64130001	0.53	4.41	0.17	-3.83	8.23	NC_022322.1	13580001	0.43	3.30	0.09	-5.29	8.59
NC_022317.1	40230001	0.43	3.38	0.12	-4.84	8.22	NC_022299.1	46840001	0.60	5.12	0.18	-3.47	8.59
NC_022298.1	92130001	0.49	4.00	0.15	-4.20	8.20	NC_022305.1	64090001	0.60	5.07	0.17	-3.51	8.58
NC_022302.1	84910001	0.58	4.89	0.19	-3.30	8.20	NC_022303.1	13170001	0.48	3.90	0.12	-4.66	8.56
NC_022295.1	61900001	0.45	3.57	0.13	-4.62	8.19	NC_022305.1	65150001	0.48	3.83	0.12	-4.73	8.56
NC_022322.1	67770001	0.56	4.72	0.18	-3.46	8.18	NC_022314.1	56540001	0.52	4.24	0.14	-4.31	8.55
NC_022302.1	84900001	0.58	4.90	0.19	-3.26	8.16	NC_022305.1	63900001	0.52	4.26	0.14	-4.27	8.53
NC_022322.1	1.08E+08	0.40	3.04	0.11	-5.12	8.15	NC_022305.1	49610001	0.62	5.31	0.19	-3.21	8.52
NC_022295.1	61850001	0.48	3.87	0.15	-4.27	8.13	NC_022293.1	1.43E+08	0.41	3.11	0.09	-5.41	8.52
NC_022310.1	34230001	0.43	3.29	0.13	-4.81	8.11	NC_022305.1	64000001	0.50	4.03	0.13	-4.49	8.52
NC_022322.1	67910001	0.56	4.72	0.19	-3.38	8.10	NC_022307.1	7900001	0.49	3.93	0.12	-4.58	8.51
NC_022298.1	92160001	0.41	3.15	0.12	-4.93	8.09	NC_022310.1	48990001	0.47	3.76	0.12	-4.72	8.47
NC_022322.1	58330001	0.48	3.81	0.15	-4.27	8.08	NC_022294.1	1.08E+08	0.53	4.34	0.15	-4.12	8.46
NC_022310.1	34220001	0.44	3.48	0.14	-4.57	8.06	NC_022305.1	64080001	0.59	5.05	0.18	-3.40	8.44
NC_022302.1	49800001	0.55	4.64	0.19	-3.41	8.06	NC_022305.1	31400001	0.43	3.35	0.10	-5.08	8.43
NC_022298.1	92170001	0.52	4.29	0.17	-3.75	8.03	NC_022305.1	65380001	0.49	3.94	0.13	-4.48	8.43
NC_022300.1	35980001	0.54	4.52	0.18	-3.51	8.03	NC_022309.1	62250001	0.48	3.84	0.12	-4.58	8.42

NC_022305.1	14680001	0.43	3.28	0.13	-4.75	8.03	NC_022305.1	64110001	0.55	4.55	0.16	-3.86	8.41
NC_022322.1	99600001	0.46	3.64	0.14	-4.38	8.03	NC_022306.1	73880001	0.59	5.05	0.18	-3.36	8.41
NC_022295.1	64140001	0.50	4.04	0.16	-3.97	8.02	NC_022305.1	63980001	0.51	4.18	0.14	-4.22	8.40
NC_022302.1	84890001	0.57	4.80	0.20	-3.19	7.99	NC_022300.1	41860001	0.43	3.31	0.10	-5.09	8.39
NC_022322.1	68170001	0.54	4.50	0.18	-3.48	7.99	NC_022305.1	65430001	0.47	3.73	0.12	-4.66	8.39
NC_022305.1	37100001	0.53	4.33	0.18	-3.65	7.99	NC_022303.1	13160001	0.48	3.84	0.13	-4.53	8.38
NC_022298.1	68420001	0.46	3.68	0.15	-4.30	7.98	NC_022322.1	42320001	0.48	3.84	0.13	-4.52	8.36
NC_022322.1	68240001	0.46	3.65	0.15	-4.30	7.95	NC_022305.1	64010001	0.50	4.09	0.14	-4.27	8.36
NC_022322.1	58600001	0.43	3.34	0.14	-4.57	7.91	NC_022305.1	63890001	0.51	4.19	0.14	-4.16	8.35
NC_022306.1	30130001	0.57	4.80	0.20	-3.09	7.89	NC_022305.1	64030001	0.50	4.06	0.14	-4.29	8.35
NC_022295.1	61860001	0.47	3.80	0.16	-4.08	7.88	NC_022322.1	8640001	0.59	5.03	0.18	-3.31	8.34
NC_022310.1	21220001	0.53	4.36	0.18	-3.49	7.86	NC_022299.1	56670001	0.56	4.66	0.17	-3.68	8.34
NC_022298.1	92180001	0.55	4.60	0.19	-3.23	7.84	NC_022306.1	73910001	0.55	4.64	0.17	-3.68	8.32
NC_022300.1	36730001	0.53	4.41	0.19	-3.41	7.82	NC_022305.1	65420001	0.45	3.56	0.12	-4.76	8.32
NC_022322.1	58590001	0.43	3.33	0.14	-4.49	7.81	NC_022305.1	64100001	0.58	4.95	0.18	-3.35	8.30
NC_022295.1	61870001	0.48	3.85	0.16	-3.94	7.79	NC_022294.1	1.08E+08	0.53	4.42	0.16	-3.86	8.28
NC_022322.1	67900001	0.55	4.62	0.20	-3.15	7.77	NC_022305.1	64070001	0.56	4.75	0.17	-3.52	8.27
NC_022302.1	50760001	0.49	3.98	0.17	-3.78	7.77	NC_022305.1	64120001	0.47	3.80	0.13	-4.46	8.25
NC_022314.1	21880001	0.46	3.64	0.16	-4.12	7.76	NC_022305.1	65370001	0.48	3.90	0.13	-4.35	8.25
NC_022304.1	25330001	0.47	3.80	0.16	-3.94	7.75	NC_022305.1	65390001	0.47	3.80	0.13	-4.45	8.25
NC_022322.1	67820001	0.54	4.53	0.19	-3.22	7.75	NC_022310.1	34180001	0.51	4.21	0.15	-4.01	8.22
NC_022297.1	22400001	0.45	3.53	0.15	-4.21	7.74	NC_022322.1	42270001	0.55	4.59	0.17	-3.62	8.21
NC_022305.1	37090001	0.51	4.14	0.18	-3.59	7.73	NC_022305.1	45260001	0.57	4.77	0.18	-3.40	8.17
NC_022295.1	61890001	0.47	3.80	0.16	-3.92	7.72	NC_022308.1	51350001	0.41	3.14	0.10	-5.01	8.15
NC_022322.1	99610001	0.45	3.60	0.16	-4.12	7.72	NC_022305.1	31410001	0.40	3.05	0.10	-5.09	8.14
NW_005101629.1	100001	0.46	3.66	0.16	-4.03	7.69	NC_022305.1	65350001	0.49	3.98	0.14	-4.15	8.13
NC_022306.1	76240001	0.43	3.38	0.15	-4.29	7.67	NC_022308.1	51340001	0.42	3.20	0.11	-4.92	8.12
NC_022322.1	58290001	0.42	3.28	0.14	-4.39	7.67	NC_022298.1	10710001	0.53	4.37	0.16	-3.74	8.12
NC_022295.1	61840001	0.45	3.58	0.16	-4.09	7.66	NC_022299.1	56640001	0.58	4.96	0.19	-3.15	8.11
NC_022296.1	90280001	0.41	3.09	0.14	-4.55	7.64	NC_022304.1	25580001	0.47	3.74	0.13	-4.36	8.11
NC_022300.1	96320001	0.50	4.05	0.18	-3.59	7.64	NC_022322.1	42280001	0.53	4.34	0.16	-3.76	8.10
NC_022300.1	36740001	0.53	4.39	0.19	-3.23	7.62	NC_022306.1	73920001	0.54	4.54	0.17	-3.56	8.10
NC_022322.1	58570001	0.40	3.01	0.13	-4.61	7.62	NC_022298.1	96980001	0.52	4.29	0.16	-3.81	8.09
NC_022313.1	61320001	0.47	3.80	0.17	-3.81	7.61	NC_022294.1	26070001	0.56	4.70	0.18	-3.40	8.09
NC_022317.1	40220001	0.41	3.08	0.14	-4.52	7.59	NC_022309.1	62220001	0.42	3.26	0.11	-4.83	8.09
NC_022295.1	61880001	0.46	3.62	0.16	-3.97	7.59	NC_022309.1	62190001	0.40	3.04	0.10	-5.05	8.09
NW_005101629.1	110001	0.43	3.39	0.15	-4.18	7.57	NC_022305.1	64020001	0.50	4.08	0.15	-4.00	8.08
NC_022322.1	69110001	0.47	3.79	0.17	-3.77	7.57	NW_005101047.1	550001	0.44	3.39	0.12	-4.67	8.06
NC_022313.1	61330001	0.47	3.74	0.17	-3.83	7.56	NC_022294.1	26170001	0.48	3.86	0.14	-4.20	8.05
NC_022322.1	67970001	0.49	3.95	0.18	-3.61	7.56	NC_022309.1	63320001	0.50	4.07	0.15	-3.98	8.05

NC_022295.1	55250001	0.41	3.14	0.14	-4.42	7.55	NC_022322.1	42120001	0.56	4.68	0.18	-3.37	8.05
NC_022300.1	9350001	0.42	3.27	0.15	-4.25	7.52	NC_022305.1	64060001	0.53	4.35	0.16	-3.70	8.05
NC_022300.1	38570001	0.52	4.32	0.19	-3.20	7.51	NC_022320.1	32430001	0.49	3.93	0.15	-4.12	8.05
NC_022302.1	50680001	0.44	3.44	0.16	-4.07	7.51	NC_022313.1	3810001	0.56	4.69	0.18	-3.36	8.05
NC_022318.1	19360001	0.50	4.05	0.18	-3.45	7.51	NC_022294.1	26060001	0.57	4.77	0.18	-3.28	8.05
NC_022322.1	67920001	0.53	4.40	0.20	-3.11	7.50	NC_022298.1	96940001	0.50	4.05	0.15	-3.99	8.04
NW_005101260.1	30001	0.41	3.08	0.14	-4.41	7.48	NC_022321.1	1630001	0.52	4.26	0.16	-3.77	8.03
NC_022304.1	25340001	0.47	3.76	0.17	-3.71	7.47	NC_022299.1	56630001	0.58	4.90	0.19	-3.09	8.00
NC_022305.1	45200001	0.46	3.65	0.17	-3.81	7.46	NC_022311.1	5370001	0.51	4.17	0.16	-3.82	7.99
NC_022305.1	75290001	0.48	3.82	0.18	-3.62	7.44	NC_022305.1	64040001	0.51	4.21	0.16	-3.77	7.98
NC_022305.1	14700001	0.41	3.12	0.15	-4.30	7.42	NC_022305.1	64050001	0.51	4.18	0.16	-3.80	7.97
NC_022313.1	61340001	0.45	3.59	0.17	-3.83	7.42	NC_022322.1	13600001	0.42	3.23	0.12	-4.74	7.97
NC_022322.1	58340001	0.46	3.60	0.17	-3.81	7.41	NC_022303.1	13150001	0.46	3.70	0.14	-4.27	7.96
NC_022302.1	50670001	0.42	3.25	0.15	-4.14	7.39	NC_022307.1	7890001	0.44	3.41	0.13	-4.54	7.95
NC_022322.1	69130001	0.40	3.06	0.15	-4.33	7.39	NC_022294.1	1.34E+08	0.43	3.32	0.12	-4.59	7.91
NC_022305.1	45210001	0.44	3.46	0.16	-3.91	7.37	NC_022322.1	42330001	0.46	3.67	0.14	-4.23	7.90
NC_022310.1	21230001	0.49	3.98	0.19	-3.37	7.35	NC_022307.1	7880001	0.41	3.16	0.12	-4.73	7.89
NC_022293.1	69810001	0.41	3.12	0.15	-4.23	7.35	NC_022299.1	46820001	0.57	4.82	0.19	-3.07	7.89
NC_022294.1	95020001	0.46	3.69	0.18	-3.64	7.34	NC_022305.1	65400001	0.42	3.23	0.12	-4.65	7.88
NC_022311.1	49860001	0.45	3.53	0.17	-3.80	7.33	NC_022305.1	64130001	0.44	3.40	0.13	-4.47	7.88
NC_022302.1	50770001	0.47	3.77	0.18	-3.54	7.31	NC_022314.1	56470001	0.48	3.91	0.15	-3.96	7.87
NC_022322.1	69100001	0.48	3.82	0.18	-3.47	7.29	NC_022309.1	63330001	0.47	3.73	0.14	-4.15	7.87
NC_022300.1	38580001	0.51	4.22	0.20	-3.07	7.29	NC_022321.1	1620001	0.49	4.00	0.16	-3.86	7.86
NC_022295.1	55260001	0.41	3.11	0.15	-4.17	7.28	NC_022294.1	1.29E+08	0.48	3.90	0.15	-3.95	7.85
NC_022295.1	64150001	0.42	3.20	0.16	-4.08	7.28	NC_022321.1	1710001	0.50	4.06	0.16	-3.77	7.83
NC_022314.1	4950001	0.49	4.01	0.19	-3.27	7.28	NC_022313.1	35430001	0.48	3.90	0.16	-3.91	7.81
NC_022302.1	840001	0.49	3.95	0.19	-3.27	7.22	NC_022311.1	5360001	0.49	4.00	0.16	-3.80	7.79
NC_022293.1	91230001	0.42	3.28	0.16	-3.93	7.21	NC_022321.1	1720001	0.49	3.97	0.16	-3.81	7.79
NC_022298.1	92120001	0.49	4.00	0.19	-3.20	7.20	NW_005101047.1	570001	0.40	3.04	0.12	-4.74	7.77
NC_022306.1	18560001	0.46	3.66	0.18	-3.53	7.19	NW_005102779.1	150001	0.42	3.25	0.13	-4.52	7.77
NC_022302.1	50750001	0.50	4.02	0.20	-3.17	7.19	NC_022310.1	53010001	0.47	3.75	0.15	-4.01	7.76
NC_022312.1	27100001	0.47	3.78	0.19	-3.38	7.16	NC_022310.1	49190001	0.51	4.13	0.17	-3.62	7.75
NC_022298.1	68440001	0.43	3.30	0.17	-3.85	7.15	NC_022313.1	60380001	0.41	3.08	0.12	-4.65	7.73
NC_022293.1	69800001	0.42	3.18	0.16	-3.93	7.10	NC_022310.1	53020001	0.45	3.50	0.14	-4.23	7.73
NC_022310.1	34270001	0.44	3.43	0.17	-3.67	7.10	NC_022306.1	73930001	0.53	4.39	0.18	-3.34	7.73

NW_005102120.1	40001	0.46	3.61	0.18	-3.48	7.09	NC_022297.1	1.03E+08	0.43	3.36	0.13	-4.36	7.72
NC_022322.1	24540001	0.44	3.47	0.18	-3.62	7.09	NC_022322.1	23480001	0.41	3.12	0.12	-4.60	7.72
NC_022310.1	21210001	0.48	3.84	0.19	-3.25	7.08	NC_022307.1	7870001	0.40	3.02	0.12	-4.70	7.71
NC_022295.1	55240001	0.42	3.25	0.17	-3.83	7.08	NC_022294.1	26310001	0.42	3.18	0.13	-4.50	7.68
NC_022302.1	49920001	0.48	3.82	0.19	-3.26	7.08	NC_022304.1	25590001	0.40	3.06	0.12	-4.61	7.67
NC_022302.1	50380001	0.45	3.60	0.18	-3.47	7.07	NC_022305.1	64140001	0.41	3.10	0.13	-4.55	7.65
NC_022312.1	27090001	0.45	3.50	0.18	-3.56	7.07	NC_022294.1	1.05E+08	0.52	4.31	0.18	-3.31	7.62
NC_022302.1	50700001	0.47	3.73	0.19	-3.33	7.07	NW_005101047.1	540001	0.43	3.31	0.14	-4.30	7.62
NW_005101260.1	50001	0.41	3.09	0.16	-3.97	7.06	NC_022294.1	26300001	0.43	3.38	0.14	-4.24	7.62
NC_022318.1	19460001	0.44	3.44	0.18	-3.59	7.03	NC_022310.1	34190001	0.50	4.06	0.17	-3.55	7.61
NC_022302.1	50690001	0.43	3.36	0.17	-3.66	7.02	NC_022306.1	62780001	0.50	4.05	0.17	-3.54	7.59
NC_022295.1	61910001	0.40	3.04	0.16	-3.96	7.00	NC_022293.1	1.43E+08	0.40	3.01	0.13	-4.57	7.58
NC_022322.1	67950001	0.48	3.84	0.20	-3.16	6.99	NC_022294.1	26160001	0.49	3.97	0.17	-3.60	7.57
NC_022322.1	69120001	0.45	3.59	0.19	-3.39	6.98	NC_022321.1	1610001	0.52	4.23	0.18	-3.33	7.56
NC_022318.1	19470001	0.48	3.89	0.20	-3.08	6.97	NC_022321.1	1730001	0.44	3.46	0.15	-4.08	7.55
NC_022318.1	19370001	0.46	3.64	0.19	-3.33	6.97	NC_022305.1	64150001	0.47	3.74	0.16	-3.81	7.55
NC_022314.1	4960001	0.45	3.53	0.18	-3.44	6.96	NC_022303.1	13140001	0.45	3.59	0.15	-3.95	7.55
NC_022322.1	24530001	0.41	3.13	0.17	-3.80	6.92	NC_022314.1	28010001	0.49	3.97	0.17	-3.57	7.54
NC_022306.1	76230001	0.43	3.35	0.18	-3.52	6.87	NC_022313.1	60370001	0.43	3.38	0.14	-4.15	7.54
NC_022302.1	50400001	0.45	3.53	0.19	-3.33	6.85	NC_022321.1	1690001	0.47	3.75	0.16	-3.78	7.54
NC_022305.1	37080001	0.47	3.77	0.20	-3.08	6.85	NC_022304.1	25920001	0.42	3.26	0.14	-4.25	7.51
NC_022322.1	58610001	0.41	3.16	0.17	-3.67	6.83	NC_022321.1	1680001	0.46	3.65	0.16	-3.83	7.48
NC_022302.1	50390001	0.45	3.53	0.19	-3.29	6.82	NC_022321.1	1700001	0.47	3.75	0.16	-3.73	7.48
NC_022310.1	24850001	0.44	3.39	0.18	-3.43	6.82	NC_022294.1	26080001	0.49	3.98	0.18	-3.47	7.45
NC_022293.1	74760001	0.41	3.09	0.17	-3.71	6.80	NC_022306.1	62770001	0.48	3.84	0.17	-3.60	7.44
NC_022314.1	28940001	0.42	3.24	0.18	-3.55	6.79	NC_022294.1	38110001	0.41	3.13	0.14	-4.30	7.44
NC_022294.1	1.24E+08	0.41	3.13	0.18	-3.65	6.77	NC_022294.1	1.05E+08	0.52	4.25	0.19	-3.17	7.42
NC_022302.1	50780001	0.43	3.38	0.19	-3.38	6.77	NC_022299.1	56680001	0.48	3.91	0.17	-3.51	7.42
NC_022304.1	25320001	0.44	3.49	0.19	-3.27	6.76	NC_022294.1	1.08E+08	0.47	3.73	0.17	-3.69	7.42
NC_022313.1	61350001	0.41	3.10	0.18	-3.65	6.75	NC_022322.1	23520001	0.45	3.49	0.15	-3.92	7.41
NC_022319.1	15320001	0.45	3.51	0.19	-3.23	6.74	NC_022306.1	22110001	0.52	4.25	0.19	-3.15	7.40
NC_022312.1	27110001	0.43	3.32	0.19	-3.40	6.72	NC_022311.1	5390001	0.49	3.96	0.18	-3.43	7.39
NC_022314.1	21890001	0.45	3.53	0.20	-3.19	6.72	NC_022310.1	34250001	0.50	4.09	0.18	-3.30	7.39
NC_022300.1	9360001	0.40	3.02	0.17	-3.68	6.70	NC_022305.1	63880001	0.49	3.92	0.18	-3.47	7.38
NC_022312.1	27120001	0.42	3.27	0.19	-3.40	6.67	NC_022294.1	1.29E+08	0.48	3.83	0.17	-3.54	7.37
NC_022295.1	68950001	0.43	3.36	0.19	-3.30	6.66	NC_022311.1	5380001	0.47	3.71	0.17	-3.66	7.37
NC_022312.1	27130001	0.41	3.10	0.18	-3.55	6.65	NC_022303.1	47860001	0.51	4.17	0.19	-3.18	7.36
NC_022294.1	1.33E+08	0.40	3.04	0.18	-3.57	6.62	NC_022310.1	34170001	0.48	3.90	0.18	-3.45	7.35

NC_022322.1	58350001	0.40	3.00	0.18	-3.60	6.60	NC_022298.1	96930001	0.47	3.78	0.17	-3.56	7.34
NC_022293.1	82020001	0.43	3.37	0.19	-3.22	6.59	NC_022309.1	71010001	0.43	3.32	0.15	-4.02	7.34
NC_022302.1	32960001	0.44	3.45	0.20	-3.12	6.57	NC_022294.1	26090001	0.47	3.73	0.17	-3.58	7.30
NC_022315.1	42390001	0.44	3.48	0.20	-3.09	6.57	NC_022314.1	53280001	0.51	4.22	0.19	-3.08	7.30
NC_022302.1	32950001	0.41	3.17	0.19	-3.40	6.57	NC_022306.1	73940001	0.49	3.97	0.18	-3.32	7.29
NC_022294.1	95030001	0.42	3.23	0.19	-3.33	6.56	NC_022305.1	65410001	0.41	3.08	0.14	-4.20	7.29
NC_022302.1	32930001	0.41	3.13	0.19	-3.40	6.53	NC_022305.1	64160001	0.48	3.84	0.18	-3.43	7.28
NC_022295.1	1.09E+08	0.44	3.47	0.20	-3.06	6.53	NC_022322.1	21050001	0.41	3.12	0.14	-4.15	7.27
NC_022300.1	42310001	0.41	3.11	0.19	-3.39	6.50	NC_022320.1	32420001	0.49	3.93	0.18	-3.34	7.27
NC_022294.1	93210001	0.44	3.39	0.20	-3.09	6.48	NC_022314.1	21960001	0.41	3.14	0.15	-4.13	7.26
NC_022293.1	83540001	0.41	3.14	0.19	-3.33	6.47	NC_022293.1	1.44E+08	0.41	3.12	0.14	-4.15	7.26
NC_022302.1	32940001	0.43	3.30	0.20	-3.16	6.46	NC_022294.1	1.05E+08	0.44	3.47	0.16	-3.79	7.26
NC_022314.1	28930001	0.42	3.18	0.19	-3.27	6.45	NC_022294.1	1.29E+08	0.48	3.91	0.18	-3.35	7.26
NC_022302.1	32970001	0.43	3.31	0.20	-3.12	6.43	NC_022321.1	1670001	0.43	3.33	0.15	-3.92	7.25
NC_022295.1	61830001	0.41	3.08	0.19	-3.31	6.38	NC_022294.1	1.08E+08	0.51	4.15	0.19	-3.10	7.25
NC_022306.1	18550001	0.41	3.08	0.19	-3.29	6.37	NC_022304.1	25950001	0.45	3.55	0.17	-3.68	7.23
NC_022308.1	73850001	0.42	3.20	0.20	-3.17	6.37	NC_022299.1	46810001	0.49	3.96	0.18	-3.28	7.23
NC_022294.1	93220001	0.42	3.25	0.20	-3.11	6.36	NC_022321.1	1650001	0.41	3.18	0.15	-4.05	7.23
NC_022294.1	1.24E+08	0.41	3.17	0.20	-3.19	6.36	NC_022304.1	50090001	0.46	3.62	0.17	-3.60	7.22
NC_022297.1	22390001	0.42	3.19	0.20	-3.15	6.34	NC_022294.1	1.05E+08	0.47	3.73	0.17	-3.49	7.22
NW_005101629.1	40001	0.40	3.04	0.19	-3.30	6.33	NC_022311.1	5350001	0.46	3.68	0.17	-3.54	7.22
NC_022313.1	5610001	0.40	3.07	0.19	-3.26	6.33	NC_022306.1	62760001	0.46	3.63	0.17	-3.58	7.21
NC_022322.1	58620001	0.42	3.18	0.20	-3.15	6.33	NC_022300.1	96310001	0.49	4.02	0.19	-3.19	7.21
NC_022293.1	79530001	0.41	3.12	0.19	-3.21	6.32	NC_022313.1	20140001	0.45	3.56	0.17	-3.64	7.19
NC_022314.1	28950001	0.41	3.13	0.20	-3.18	6.31	NC_022310.1	49200001	0.42	3.28	0.16	-3.91	7.19
NC_022294.1	1.1E+08	0.40	3.03	0.19	-3.28	6.31	NC_022304.1	25930001	0.43	3.29	0.16	-3.89	7.19
NC_022307.1	22930001	0.42	3.19	0.20	-3.10	6.29	NC_022304.1	25570001	0.47	3.78	0.18	-3.39	7.17
NC_022310.1	24860001	0.41	3.15	0.20	-3.12	6.27	NC_022295.1	21930001	0.48	3.86	0.18	-3.30	7.17
NC_022299.1	4250001	0.41	3.09	0.20	-3.10	6.20	NC_022305.1	45000001	0.46	3.60	0.17	-3.55	7.15
NC_022322.1	95090001	0.41	3.11	0.20	-3.06	6.17	NC_022314.1	21970001	0.42	3.25	0.16	-3.88	7.13
NC_022293.1	91240001	0.40	3.04	0.20	-3.13	6.16	NC_022310.1	53030001	0.45	3.50	0.17	-3.62	7.12
NC_022293.1	79510001	0.41	3.10	0.20	-3.05	6.15	NC_022322.1	8660001	0.50	4.03	0.19	-3.09	7.12
NC_022296.1	7620001	0.40	3.04	0.20	-3.08	6.12	NC_022302.1	49050001	0.41	3.11	0.15	-4.01	7.12
							NC_022314.1	21990001	0.44	3.47	0.17	-3.64	7.11
							NC_022310.1	34160001	0.49	3.97	0.19	-3.14	7.10
							NC_022302.1	49060001	0.41	3.18	0.15	-3.92	7.10
							NC_022304.1	50100001	0.47	3.76	0.18	-3.33	7.09
							NC_022304.1	25960001	0.44	3.42	0.17	-3.67	7.09
							NC_022314.1	21950001	0.44	3.47	0.17	-3.61	7.08
							NC_022304.1	25940001	0.44	3.43	0.17	-3.65	7.08
							NC_022304.1	50110001	0.47	3.81	0.18	-3.27	7.08

							NC_022321.1	1740001	0.43	3.36	0.16	-3.70	7.06	
							NC_022316.1	2810001	0.40	3.07	0.15	-3.99	7.06	
							NC_022310.1	34260001	0.47	3.78	0.18	-3.28	7.05	
							NC_022313.1	35440001	0.47	3.73	0.18	-3.31	7.04	
							NC_022294.1	1.05E+08	0.47	3.73	0.18	-3.29	7.02	
							NC_022305.1	44990001	0.46	3.61	0.18	-3.41	7.02	
							NC_022303.1	47880001	0.48	3.88	0.19	-3.14	7.01	
							NC_022309.1	62210001	0.44	3.42	0.17	-3.59	7.01	
							NC_022314.1	27970001	0.46	3.63	0.18	-3.37	7.00	
							NC_022305.1	62970001	0.47	3.74	0.19	-3.25	6.98	
							NC_022304.1	50120001	0.47	3.76	0.19	-3.22	6.98	
							NC_022321.1	1660001	0.40	3.03	0.15	-3.93	6.97	
							NC_022295.1	21920001	0.45	3.57	0.18	-3.39	6.96	
							NC_022309.1	34250001	0.41	3.17	0.16	-3.79	6.96	
							NC_022309.1	65900001	0.40	3.07	0.16	-3.87	6.94	
							NC_022322.1	23530001	0.45	3.53	0.18	-3.39	6.92	
							NC_022322.1	2910001	0.42	3.22	0.17	-3.70	6.91	
							NC_022296.1	65610001	0.43	3.30	0.17	-3.62	6.91	
							NC_022322.1	42340001	0.46	3.66	0.19	-3.25	6.91	
							NC_022306.1	62750001	0.44	3.44	0.18	-3.46	6.90	
							NC_022309.1	71000001	0.41	3.09	0.16	-3.81	6.90	
							NC_022294.1	26180001	0.41	3.07	0.16	-3.81	6.88	
							NC_022303.1	47890001	0.47	3.72	0.19	-3.16	6.88	
							NC_022314.1	56460001	0.46	3.68	0.19	-3.19	6.87	
							NC_022310.1	34270001	0.44	3.43	0.18	-3.43	6.86	
							NC_022296.1	1.15E+08	0.41	3.12	0.16	-3.74	6.85	
							NC_022309.1	65890001	0.40	3.05	0.16	-3.79	6.84	
							NC_022306.1	62720001	0.46	3.61	0.19	-3.22	6.83	
							NC_022296.1	1.15E+08	0.43	3.37	0.18	-3.41	6.78	
							NC_022304.1	50130001	0.45	3.54	0.19	-3.20	6.75	
							NC_022310.1	53000001	0.44	3.46	0.18	-3.28	6.75	
							NC_022306.1	62740001	0.44	3.48	0.19	-3.25	6.73	
							NC_022302.1	47810001	0.46	3.67	0.19	-3.06	6.73	
							NC_022305.1	45030001	0.44	3.42	0.18	-3.28	6.71	
							NC_022314.1	33330001	0.43	3.32	0.18	-3.38	6.70	
							NC_022293.1	1.44E+08	0.40	3.06	0.17	-3.63	6.70	
							NC_022306.1	32100001	0.42	3.25	0.18	-3.45	6.69	
							NC_022293.1	1.44E+08	0.42	3.22	0.18	-3.46	6.68	
							NC_022296.1	1.15E+08	0.44	3.46	0.19	-3.22	6.68	
							NC_022311.1	25810001	0.45	3.53	0.19	-3.14	6.67	

							NC_022304.1	25970001	0.42	3.24	0.18	-3.43	6.66
							NC_022314.1	33340001	0.43	3.31	0.18	-3.35	6.66
							NC_022294.1	1.29E+08	0.45	3.56	0.19	-3.08	6.64
							NC_022304.1	50080001	0.41	3.17	0.18	-3.45	6.62
							NC_022305.1	44980001	0.43	3.37	0.19	-3.25	6.62
							NC_022306.1	73950001	0.42	3.18	0.18	-3.43	6.61
							NC_022298.1	96920001	0.42	3.28	0.18	-3.32	6.60
							NC_022305.1	65340001	0.41	3.15	0.18	-3.42	6.57
							NC_022305.1	65550001	0.41	3.10	0.18	-3.47	6.57
							NC_022309.1	34300001	0.40	3.03	0.17	-3.52	6.55
							NC_022314.1	33350001	0.41	3.08	0.18	-3.47	6.55
							NC_022305.1	45040001	0.42	3.24	0.18	-3.31	6.54
							NC_022322.1	1.16E+08	0.44	3.39	0.19	-3.13	6.52
							NC_022303.1	93420001	0.40	3.00	0.17	-3.52	6.52
							NC_022293.1	1.43E+08	0.40	3.03	0.17	-3.48	6.52
							NC_022311.1	26360001	0.43	3.32	0.19	-3.19	6.51
							NC_022294.1	1.29E+08	0.41	3.08	0.18	-3.37	6.45
							NC_022297.1	84260001	0.40	3.04	0.18	-3.40	6.44
							NC_022299.1	56690001	0.41	3.15	0.18	-3.27	6.42
							NC_022305.1	45010001	0.42	3.19	0.19	-3.22	6.42
							NC_022309.1	34290001	0.41	3.15	0.18	-3.26	6.41
							NC_022309.1	34280001	0.41	3.12	0.18	-3.29	6.41
							NC_022295.1	61140001	0.41	3.18	0.19	-3.21	6.39
							NC_022310.1	48980001	0.42	3.23	0.19	-3.14	6.37
							NC_022306.1	79430001	0.42	3.23	0.19	-3.14	6.36
							NC_022305.1	45020001	0.42	3.28	0.19	-3.06	6.34
							NC_022296.1	65620001	0.40	3.06	0.18	-3.27	6.32
							NC_022309.1	62200001	0.42	3.19	0.19	-3.12	6.31
							NC_022305.1	63570001	0.40	3.02	0.18	-3.28	6.30
							NC_022310.1	34280001	0.41	3.15	0.19	-3.13	6.28
							NC_022305.1	45050001	0.40	3.07	0.19	-3.20	6.27
							NC_022295.1	21910001	0.40	3.07	0.19	-3.20	6.27
							NC_022294.1	26100001	0.41	3.11	0.19	-3.15	6.26
							NC_022297.1	84270001	0.40	3.04	0.19	-3.22	6.25
							NC_022309.1	65880001	0.40	3.05	0.19	-3.20	6.25
							NC_022296.1	65600001	0.41	3.08	0.19	-3.14	6.23

Supplementary table 5 Candidate genes of selective sweep.

Alleles frequency difference of DBG	Alleles frequency difference of IMCG	Genetic differentiation	Regions of low heterozygosity and high genetic differentiation from DBG	Regions of low heterozygosity and high genetic differentiation from IMCG

ACOT9	ABHD15	ACAP1	ACAP1	AK3
ADIG	AMER1	ADAM10	ADAM10	AKTIP
ANK2	ANKRD13B	ADIG	ADIG	ALKBH1
ANKRD17	APTX	AK3	ANK2	C6H4orf22
ARHGEF17	BCL2L12	AKTIP	ATP11C	CCDC136
BCL2L12	BRPF3	ALKBH1	ATP6V0D1	CCDC160
BIRC6	C1H3orf33	ANK2	BAI1	CDC37L1
C11H2orf68	C6H4orf22	ARID1B	BBS5	CELF2
C25H16orf13	CALU	ATL1	C19H17orf74	CFHR5
CACNB2	CCDC136	ATP11C	CACNB2	CHUK
CCDC64	CCDC152	ATP6	CCDC64	CIAPIN1
CCDC73	CELF2	ATP8	CGNL1	COQ9
CNBP	CFHR2	BBS5	CLN5	CRB1
EPB41L5	CFHR5	BPI	CNBP	CSE1L
FAM195A	CHIC2	C6H4orf22	CNTN5	CWF19L1
FAM3A	CSE1L	CA7	CPNE1	DACH2
FBXL16	DDX27	CC2D1B	CRYBA4	DDAH1
G6PD	DIO3	CDC37L1	CRYBB1	DMD
GCN1L1	DTNBP1	CDH16	DENND1A	DOK4
GGCX	EGLN1	CDK19	DIP2A	ELOVL2
GPR37L1	F13B	CHGB	DIP2C	EMC1
GRAMD3	FARP1	CHMP3	DLGAP4	ERLIN1
IKBKG	FGF12	CIITA	DNAJC18	ETV5
IRF3	GIT1	CLEC16A	DRP2	F13B
KDR	GPC5	CNBP	DSN1	FARP1
LATS2	HEPH	COX1	ECSCR	FAT1
LMOD1	IDH1	COX2	EFCC1	FGF5
LOC102168445	IQCE	COX3	EHD2	FLNC
LOC102169755	IRF3	CPN1	EIF5A	GATAD1
LOC102171901	JARID2	CPNE1	FASTKD1	GIGYF2
LOC102174442	KANK1	CRADD	FAT3	HMBOX1
LOC102174728	KLHL15	CSE1L	FBXL3	HSD11B2
LOC102178382	KLHL24	CYTB	GCN1L1	IFNLR1
LOC102180475	LAS1L	DNAJC18	GLTSCR1	INO80
LOC102180747	LFNG	DNMBP	GPC3	IQCE
LOC102187329	LMOD1	DRP2	GPS2	KDM4C
LOC102187462	LOC102169221	DSG2	HSD11B2	KLHL15
LOC102187584	LOC102169338	DSG3	IGSF21	LFNG
LOC102187747	LOC102169755	ECSCR	IQGAP1	LMF2
LOC102189985	LOC102170078	EDC4	KCTD10	LOC102171894
LOC102190823	LOC102170346	EIF5A	KCTD11	LOC102173932
MAT2A	LOC102175111	ELOVL2	KCTD19	LOC102175291

MID2	LOC102175310	FARP1	KDM1A	LOC102175359
MSI1	LOC102175900	FASTKD1	KDM3A	LOC102176407
NAGK	LOC102175937	FRMD4A	KIAA1755	LOC102176507
NOSIP	LOC102175971	FRMPD3	KLHL41	LOC102176839
PAIP2B	LOC102177261	GLDC	LACTBL1	LOC102178624
PCBP3	LOC102177547	GPC3	LOC102169041	LOC102179113
PLA2G1B	LOC102180113	GPS2	LOC102169373	LOC102179840
POLN	LOC102183950	IFNLR1	LOC102169660	LOC102180113
PRDM2	LOC102184002	IL22RA1	LOC102171901	LOC102180722
PRDX4	LOC102184889	KCTD11	LOC102173128	LOC102181499
PRR12	LOC102186596	KDM3A	LOC102174442	LOC102181835
PRRG2	LOC102188087	KDM4B	LOC102174728	LOC102187904
PTPN7	LOC102188589	KDM4C	LOC102178382	LOC102188899
PTPRN2	LOC102188699	KIAA1755	LOC102178836	LOC102189440
PXN	LOC102188965	KLHL41	LOC102179310	LOC102189935
PYGM	LOC102190928	LCAT	LOC102180340	LOC102190889
RAB35	LOC102191239	LFNG	LOC102181054	LOC102190928
RALGAPB	LRRC36	LIPC	LOC102184370	LOC102190988
RASGRP2	MORC4	LOC102169849	LOC102184932	LOC102191670
RHBDL1	NAP1L4	LOC102170024	LOC102185208	LRRC36
RHOT2	NCBP2	LOC102171175	LOC102186320	MAFG
RNF181	NIPAL2	LOC102171894	LOC102187329	MCF2L2
RPLP0	OPN1SW	LOC102174442	LOC102189985	MIOX
RRAS	OXTR	LOC102175219	LOC102189991	MYADML2
SCAF1	PIKFYVE	LOC102175291	LOC102190081	NCBP2
SCML2	PPP6R2	LOC102175415	LOC102190542	NEDD4
6-Sep	PRR12	LOC102177076	LOC102190811	NOTUM
SIRT4	PTH2R	LOC102177356	LOC102190823	PARD3B
SORCS3	RAD18	LOC102178836	LRP2	PEX1
SPATA5	RRAS	LOC102179449	LRRC36	PHACTR4
STUB1	RRM1	LOC102179840	MAVS	PIGK
TIMM17A	RYR3	LOC102181499	MBNL3	POLR2C
TMEM150A	SBF1	LOC102183378	MID2	POLR3F
TRNAC-ACA	SCAF1	LOC102184370	MROH8	PPP6R2
TRNAG-CCC	SENP5	LOC102186029	MSI1	PRND
TSC22D3	SEPP1	LOC102187329	MYCBP2	PRNP
TTC27	SGOL1	LOC102188681	MYO1H	PRTG
USP39	SLC15A2	LOC102189991	MZB1	PYCR1
VAMP5	SLC22A18	LOC102191295	NEURL4	RBBP9
VAMP8	SLC33A1	LOC102191670	NFS1	RBL2
WDR24	SPRTN	LUZP6	NKRF	RFX7
WDR90	STAU1	MAVS	NLGN2	RYBP

WFIKKN1	STIM1	MBNL3	PABPC1	SBF1
ZNF385B	TAB1	MCF2	PAIP2	SEC23B
	TAOK1	MCM8	PAIP2B	SENP5
	TCF12	MMGT1	PCBP3	SETD3
	TIMM17A	MROH8	PLA2G1B	SETMAR
	TP53I13	MTMR7	PLD2	SLIRP
	TPPP3	MTPN	PLEKHG4	SNW1
	TRNAE-UUC	MZB1	PLSCR3	ST3GAL1
	TRNAG-CCC	ND1	PRDM2	STAU1
	TRNAP-AGG	ND2	PRND	SUMF1
	TTYH3	ND3	PROB1	SYNM
	ZC4H2	ND4	PSMB6	TPPP3
	ZDHHC1	ND4L	PTPRN2	TRNAA-UGC
		ND5	PXN	TRNAE-UUC
		ND6	RALGAPB	TRNAW-CCA
		NEDD4	RB1CC1	TTC23
		NEK1	RBL1	TTYH3
		NEURL4	RBM12	UBR4
		NFS1	RBM39	UNC13C
		NLGN2	ROMO1	ZBTB16
		NRN1L	RPLP0	ZDHHC1
		NUTF2	SACM1L	ZNF280D
		ORC1	SAMHD1	
		PABPC1	6-Sep	
		PAIP2	SH3TC2	
		PANK2	SIRT4	
		PDP2	SLC23A1	
		PIGK	SLC9A5	
		PLSCR3	SOGA1	
		PPAPDC2	SPATA24	
		PRND	SPATA5	
		PRNP	SPEM1	
		PROB1	STARD13	
		PRPF18	TLDC2	
		PSKH1	TMEM256	
		PSMB10	TMEM27	
		PTPRS	TMEM95	
		RALGAPB	TNK1	
		RBL1	TPPP3	
		RBL2	TRNAC-ACA	
		RBM12	TRNAE-UUC	
		RBM39	TRNAG-CCC	

		RCL1	TRNAR-ACG	
		RNF103	TRNASTOP-CUA	
		RNF24	TRNAW-CCA	
		ROMO1	TRNAY-AUA	
		SAMHD1	TSC22D3	
		SETMAR	UBE3B	
		SH3TC2	WARS2	
		SLC12A4	ZDHHC1	
		SLC23A1	ZMYND11	
		SLIRP		
		SNW1		
		SOGA1		
		SPAG4		
		SPATA24		
		SPATA6L		
		STAU1		
		SUMF1		
		THAP11		
		TLDC2		
		TMEM106B		
		TMEM256		
		TMEM95		
		TNK1		
		TRERF1		
		TRIM34		
		TRIM6		
		TRMT6		
		TRNAC-ACA		
		TRNAE-UUC		
		TRNAS-AGA		
		TRNAW-CCA		
		TTR		
		TTYH3		
		UHRF2		
		UNC13C		
		VWDE		
		ZFYVE9		
		ZNF280D		

Supplementary table 6 The result lists of GO annotation

**Alleles frequency difference of DBG**			
**GO_accession**	**Description**	**Over_represented**	**Gene_names**

		_pValue	
GO:0004645	phosphorylase activity	0.0015924	SORCS3,PYGM
GO:0008184	glycogen phosphorylase activity	0.0015924	SORCS3,PYGM
GO:0005375	copper ion transmembrane transporter activity	0.006132	CACNB2,POLN,ANK2
GO:0035434	copper ion transmembrane transport	0.006132	ANK2,POLN,CACNB2
GO:0004725	protein tyrosine phosphatase activity	0.0067943	SORCS3,PTPRN2,LOC102168445,PTPN7
GO:0043903	regulation of symbiosis, encompassing mutualism through parasitism	0.0083609	SORCS3,TTC27,RALGAPB,ANKRD17
GO:0005185	neurohypophyseal hormone activity	0.0085707	PAIP2B,CCDC64,EPB41L5
GO:0004842	ubiquitin-protein ligase activity	0.0090962	RNF181,ARHGEF17,MID2,BIRC6,SPATA5,ANK2,STUB1
GO:0019787	small conjugating protein ligase activity	0.0090962	ANK2,STUB1,SPATA5,BIRC6,ARHGEF17,MID2,RNF181
GO:0008488	gamma-glutamyl carboxylase activity	0.009296	ZNF385B,GGCX
GO:0017187	peptidyl-glutamic acid carboxylation	0.009296	ZNF385B,GGCX
GO:0018200	peptidyl-glutamic acid modification	0.009296	GGCX,ZNF385B
GO:0018214	protein carboxylation	0.009296	ZNF385B,GGCX
GO:0016567	protein ubiquitination	0.0094898	STUB1,ANK2,MID2,ARHGEF17
GO:0008140	cAMP response element binding protein binding	0.0098946	CACNB2
GO:0051289	protein homotetramerization	0.0098946	CACNB2
GO:0015204	urea transmembrane transporter activity	0.010052	BIRC6,SEPT6
GO:0015840	urea transport	0.010052	BIRC6,SEPT6
GO:0019755	one-carbon compound transport	0.010052	BIRC6,SEPT6
GO:0042887	amide transmembrane transporter activity	0.010052	SEPT6,BIRC6
GO:0071918	urea transmembrane transport	0.010052	BIRC6,SEPT6
GO:0016881	acid-amino acid ligase activity	0.012008	ARHGEF17,BIRC6,ANK2,STUB1,RNF181,MID2,SPATA5,CACNB2,SORCS3
GO:0016049	cell growth	0.012089	PTPRN2,G6PD
GO:0032446	protein modification by small protein conjugation	0.013525	STUB1,ANK2,MID2,ARHGEF17
GO:0070647	protein modification by small protein conjugation or removal	0.013525	MID2,ARHGEF17,STUB1,ANK2
GO:0006825	copper ion transport	0.018294	POLN,CACNB2,ANK2
GO:0016433	rRNA (adenine) methyltransferase activity	0.018688	C25H16orf13,SORCS3

GO:0004721	phosphoprotein phosphatase activity	0.02089	LOC102168445,PTPRN2,PTPN7,SORCS3
GO:0006470	protein dephosphorylation	0.022032	PTPN7,PTPRN2,LOC102168445
GO:0034599	cellular response to oxidative stress	0.024025	PTPRN2,PCBP3
GO:0060341	regulation of cellular localization	0.026057	TSC22D3,GRAMD3,CACNB2
GO:0050792	regulation of viral process	0.033771	SORCS3,RALGAPB,ANKRD17
GO:0004357	glutamate-cysteine ligase activity	0.034023	SORCS3,CACNB2
GO:0051225	spindle assembly	0.034949	USP39
GO:0015556	C4-dicarboxylate transmembrane transporter activity	0.035447	ANK2
GO:0032880	regulation of protein localization	0.037223	TSC22D3,CACNB2,GRAMD3
GO:0051223	regulation of protein transport	0.037223	CACNB2,GRAMD3,TSC22D3
GO:0070201	regulation of establishment of protein localization	0.037223	TSC22D3,GRAMD3,CACNB2
GO:0009881	photoreceptor activity	0.038817	BIRC6
GO:0009882	blue light photoreceptor activity	0.038817	BIRC6
GO:0071949	FAD binding	0.038817	BIRC6
GO:0004564	beta-fructofuranosidase activity	0.040161	PTPRN2,LOC102171901
GO:0004575	sucrose alpha-glucosidase activity	0.040161	PTPRN2,LOC102171901
GO:0005750	mitochondrial respiratory chain complex III	0.0406	ZNF385B,PTPRN2
GO:0006122	mitochondrial electron transport, ubiquinol to cytochrome c	0.0406	ZNF385B,PTPRN2
GO:0045275	respiratory chain complex III	0.0406	PTPRN2,ZNF385B
GO:0009832	plant-type cell wall biogenesis	0.041948	G6PD
GO:0010215	cellulose microfibril organization	0.041948	G6PD
GO:0070726	cell wall assembly	0.041948	G6PD
GO:0071668	plant-type cell wall assembly	0.041948	G6PD
GO:0016879	ligase activity, forming carbon-nitrogen bonds	0.042266	ARHGEF17,STUB1,ANK2,GCN1L1,BIRC6,MID2,RNF181,SORCS3,CACNB2,SPATA5
GO:0000151	ubiquitin ligase complex	0.044624	STUB1,ANK2,SPATA5,MID2,RNF181
GO:0004336	galactosylceramidase activity	0.04583	LOC102171901
GO:0006677	glycosylceramide metabolic process	0.04583	LOC102171901
GO:0006681	galactosylceramide metabolic process	0.04583	LOC102171901
GO:0006683	galactosylceramide catabolic process	0.04583	LOC102171901
GO:0006687	glycosphingolipid metabolic process	0.04583	LOC102171901
GO:0019374	galactolipid metabolic process	0.04583	LOC102171901
GO:0019376	galactolipid catabolic process	0.04583	LOC102171901
GO:0019377	glycolipid catabolic process	0.04583	LOC102171901
GO:0030149	sphingolipid catabolic process	0.04583	LOC102171901
GO:0046466	membrane lipid catabolic process	0.04583	LOC102171901

GO:0046477	glycosylceramide catabolic process	0.04583	LOC102171901
GO:0046479	glycosphingolipid catabolic process	0.04583	LOC102171901
GO:0046514	ceramide catabolic process	0.04583	LOC102171901
GO:0004026	alcohol O-acetyltransferase activity	0.046097	EPB41L5
GO:0034318	alcohol O-acyltransferase activity	0.046097	EPB41L5
GO:0042597	periplasmic space	0.046963	TTC27,SORCS3,G6PD
GO:0032505	reproduction of a single-celled organism	0.04956	CCDC64,TSC22D3
GO:0043093	cytokinesis by binary fission	0.04956	CCDC64,TSC22D3
**Alleles frequency difference of IMCG**			
**GO_accession**	**Description**	**Over_represented_pValue**	**Gene_names**
GO:0004748	ribonucleoside-diphosphate reductase activity, thioredoxin disulfide as acceptor	0.00084853	LOC102184002,CELF2,RRM1
GO:0016728	oxidoreductase activity, acting on CH or CH2 groups, disulfide as acceptor	0.00084853	RRM1,LOC102184002,CELF2
GO:0016725	oxidoreductase activity, acting on CH or CH2 groups	0.0017932	RRM1,CELF2,LOC102184002
GO:0031109	microtubule polymerization or depolymerization	0.0036354	RYR3,C6H4orf22
GO:0031110	regulation of microtubule polymerization or depolymerization	0.0036354	RYR3,C6H4orf22
GO:0016730	oxidoreductase activity, acting on iron-sulfur proteins as donors	0.0066685	GPC5,F13B,LOC102175937,FARP1
GO:0006542	glutamine biosynthetic process	0.010213	STIM1,GPC5
GO:0009291	unidirectional conjugation	0.012759	CELF2,GPC5
GO:0019953	sexual reproduction	0.013741	TPPP3,TAB1,GPC5,JARID2,SBF1,LOC102188589,SLC22A18,CCDC136
GO:0006891	intra-Golgi vesicle-mediated transport	0.013904	FGF12,KLHL24,GPC5,C6H4orf22,ZC4H2,CELF2
GO:0016307	phosphatidylinositol phosphate kinase activity	0.01431	CALU,PIKFYVE
GO:0007131	reciprocal meiotic recombination	0.020643	SLC33A1,C6H4orf22,TAOK1
GO:0035825	reciprocal DNA recombination	0.020643	SLC33A1,C6H4orf22,TAOK1
GO:0000003	reproduction	0.021444	JARID2,TAB1,CELF2,AMER1,KANK1,C6H4orf22,SGOL1,TCF12,TPPP3,GPC5,TAOK1,SLC22A18,CCDC136,DTNBP1,SBF1,SLC33A1,LOC102188589
GO:0006304	DNA modification	0.021621	CELF2,LOC102184002,LOC102175310,GPC5,JARID2,C6H4orf22
GO:0007126	meiosis	0.02174	TAOK1,C6H4orf22,SGOL1,SLC33A1
GO:0051321	meiotic cell cycle	0.02174	SGOL1,SLC33A1,TAOK1,C6H4orf22

GO:0006231	dTMP biosynthetic process	0.022593	HEPH,BCL2L12
GO:0009157	deoxyribonucleoside monophosphate biosynthetic process	0.022593	BCL2L12,HEPH
GO:0009176	pyrimidine deoxyribonucleoside monophosphate metabolic process	0.022593	BCL2L12,HEPH
GO:0009177	pyrimidine deoxyribonucleoside monophosphate biosynthetic process	0.022593	HEPH,BCL2L12
GO:0009221	pyrimidine deoxyribonucleotide biosynthetic process	0.022593	HEPH,BCL2L12
GO:0009263	deoxyribonucleotide biosynthetic process	0.022593	HEPH,BCL2L12
GO:0009265	2'-deoxyribonucleotide biosynthetic process	0.022593	BCL2L12,HEPH
GO:0042083	5,10-methylenetetrahydrofolate-dependent methyltransferase activity	0.022593	BCL2L12,HEPH
GO:0046073	dTMP metabolic process	0.022593	HEPH,BCL2L12
GO:0046385	deoxyribose phosphate biosynthetic process	0.022593	HEPH,BCL2L12
GO:0030255	protein secretion by the type IV secretion system	0.026906	GPC5,STIM1
GO:0044097	secretion by the type IV secretion system	0.026906	STIM1,GPC5
GO:0006323	DNA packaging	0.028398	TCF12,LOC102175937,KANK1,GPC5,RAD18,NAP1L4
GO:0005135	interleukin-3 receptor binding	0.030544	LOC102175310
GO:0000781	chromosome, telomeric region	0.030945	GPC5,RAD18
GO:0009162	deoxyribonucleoside monophosphate metabolic process	0.032098	HEPH,BCL2L12
GO:0009292	genetic transfer	0.033612	GPC5,CELF2
GO:0005956	protein kinase CK2 complex	0.037469	FARP1,C6H4orf22
GO:0016278	lysine N-methyltransferase activity	0.037955	LOC102188965,GPC5,ZC4H2,CFHR5
GO:0016279	protein-lysine N-methyltransferase activity	0.037955	LOC102188965,GPC5,ZC4H2,CFHR5
GO:0018024	histone-lysine N-methyltransferase activity	0.037955	LOC102188965,GPC5,ZC4H2,CFHR5
GO:0042054	histone methyltransferase activity	0.037955	ZC4H2,CFHR5,LOC102188965,GPC5
GO:0006541	glutamine metabolic process	0.039132	GPC5,STIM1
GO:0032886	regulation of microtubule-based process	0.039459	RYR3,C6H4orf22
GO:0070507	regulation of microtubule cytoskeleton organization	0.039459	RYR3,C6H4orf22
GO:0004640	phosphoribosylanthranilate isomerase	0.040755	TAB1

	activity		
GO:0005971	ribonucleoside-diphosphate reductase complex	0.041865	CELF2
GO:0007050	cell cycle arrest	0.042139	LOC102170078,TCF12,GPC5
GO:0019867	outer membrane	0.044197	GPC5,FGF12,JARID2,STIM1,HEPH,STAU1,ZC4H2,CELF2,C6H4orf22,LOC102188965
GO:0042626	ATPase activity, coupled to transmembrane movement of substances	0.0443	C6H4orf22,KANK1,CCDC136,GPC5,TAOK1,IQCE,SLC15A2
GO:0043492	ATPase activity, coupled to movement of substances	0.0443	TAOK1,C6H4orf22,KANK1,GPC5,CCDC136,SLC15A2,IQCE
GO:0000070	mitotic sister chromatid segregation	0.045904	LOC102175937
GO:0000796	condensin complex	0.045904	LOC102175937
GO:0000819	sister chromatid segregation	0.045904	LOC102175937
GO:0007076	mitotic chromosome condensation	0.045904	LOC102175937
GO:0000786	nucleosome	0.04891	GPC5,TPPP3,SBF1
GO:0032993	protein-DNA complex	0.04891	SBF1,TPPP3,GPC5
GO:0006520	cellular amino acid metabolic process	0.049409	LOC102188589,SLC22A18,GIT1,DTNBP1,NAP1L4,TCF12,STIM1,GPC5,LOC102188965,RYR3,C6H4orf22,KANK1,CELF2,SLC15A2,TAB1,FGF12,JARID2
GO:0007127	meiosis I	0.049581	SLC33A1,C6H4orf22,TAOK1
**Genetic differentiation**			
**GO_accession**	**Description**	**Over_represented_pValue**	**Gene_names**
GO:0008704	5-carboxymethyl-2-hydroxymuconate delta-isomerase activity	0.0013555	PIGK,CRADD
GO:0016812	hydrolase activity, acting on carbon-nitrogen (but not peptide) bonds, in cyclic amides	0.0020763	ACAP1,KDM4C
GO:0045321	leukocyte activation	0.0028889	LOC102169849,CDK19,CLEC16A
GO:0022884	macromolecule transmembrane transporter activity	0.0042958	KDM4C,ARID1B,UNC13C,FRMD4A
GO:0004531	deoxyribonuclease II activity	0.0051302	C6H4orf22,FRMD4A
GO:0016889	endodeoxyribonuclease activity, producing 3'-phosphomonoesters	0.0051302	C6H4orf22,FRMD4A
GO:0007156	homophilic cell adhesion	0.0069765	DSG3,DSG2,LOC102169849,CDH16
GO:0046907	intracellular transport	0.0093982	C6H4orf22,SPATA6L,ATL1,CHMP3,MCM8,KDM4C,CDC37L1,UHRF2,FRMPD3,ADAM10,NEK1,STAU1,ND5,LOC102170024,UNC13C,NEDD4,FRM

			D4A,CSE1L,TRIM6,ANK2,IL22RA1,MBNL3,CDK19,LOC102169849,MTMR7,LOC102179840,PIGK,GPC3,DNAJC18,PDP2,CRADD,FARP1
GO:0006605	protein targeting	0.0098168	ATL1,ND5,LOC102170024,CHMP3,CRADD,LOC102179840,PIGK,ADAM10,C6H4orf22,MBNL3,UHRF2,KDM4C,CDC37L1
GO:0033615	mitochondrial proton-transporting ATP synthase complex assembly	0.011774	SETMAR,LOC102169849
GO:0030683	evasion or tolerance by virus of host immune response	0.011854	UNC13C,ADAM10,KDM4C,GPC3
GO:0051649	establishment of localization in cell	0.012321	CDC37L1,KDM4C,UHRF2,CHMP3,MCM8,ATL1,C6H4orf22,SPATA6L,NEDD4,UNC13C,STAU1,NEK1,LOC102170024,ND5,ARID1B,ADAM10,FRMPD3,MBNL3,LOC102169849,CDK19,IL22RA1,TRIM6,ANK2,FRMD4A,CSE1L,CIITA,FARP1,GPC3,CRADD,PDP2,DNAJC18,MTMR7,LOC102179840,PIGK
GO:0019385	methanogenesis, from acetate	0.013811	ORC1,CRADD
GO:0005787	signal peptidase complex	0.014576	CDK19,ZFYVE9,NEDD4,RBM39,C6H4orf22,ARID1B
GO:0006465	signal peptide processing	0.014576	RBM39,ARID1B,C6H4orf22,ZFYVE9,CDK19,NEDD4
GO:0033227	dsRNA transport	0.014771	FRMD4A,KDM4C
GO:0051032	nucleic acid transmembrane transporter activity	0.014771	KDM4C,FRMD4A
GO:0051033	RNA transmembrane transporter activity	0.014771	KDM4C,FRMD4A
GO:0046649	lymphocyte activation	0.016418	CLEC16A,LOC102169849
GO:0051641	cellular localization	0.017894	NEDD4,UNC13C,ND5,LOC102170024,NEK1,STAU1,RALGAPB,FRMPD3,ARID1B,ADAM10,UHRF2,KDM4C,CDC37L1,ATL1,MCM8,CHMP3,SPATA6L,C6H4orf22,FARP1,CIITA,PDP2,DNAJC18,CRADD,GPC3,LOC102179840,PIGK,MTMR7,MBNL3,CDK19,LOC102169849,TRIM6,ANK2,IL22RA1,CSE1L,FRMD4A
GO:0020012	evasion or tolerance of host immune response	0.018435	UNC13C,ADAM10,KDM4C,GPC3
GO:0051805	evasion or tolerance of immune response of other organism involved in symbiotic interaction	0.018435	ADAM10,KDM4C,UNC13C,GPC3
GO:0052564	response to immune response of other organism involved in symbiotic interaction	0.018435	GPC3,ADAM10,KDM4C,UNC13C

GO:0052572	response to host immune response	0.018435	GPC3,UNC13C,KDM4C,ADAM10
GO:0006839	mitochondrial transport	0.018688	ATL1,UNC13C,SPATA6L,ADAM10,CDC37L1,KDM4C
GO:0031090	organelle membrane	0.01911	COX2,LUZP6,LOC102186029,SH3TC2,FARP1,CIITA,DNAJC18,GPC3,PIGK,RBM39,LOC102169849,CDK19,ANK2,RNF103,SAMHD1,FRMD4A,NEDD4,SETMAR,UNC13C,LOC102170024,NEK1,ORC1,ADAM10,ARID1B,TRERF1,ZFYVE9,LIPC,RNF24,IFNLR1,KDM4C,AK3,CDC37L1,ATL1,GLDC,SPATA6L,C6H4orf22
GO:0043461	proton-transporting ATP synthase complex assembly	0.019872	SETMAR,LOC102169849
GO:0070071	proton-transporting two-sector ATPase complex assembly	0.019872	SETMAR,LOC102169849
GO:0070272	proton-transporting ATP synthase complex biogenesis	0.019872	SETMAR,LOC102169849
GO:0044446	intracellular organelle part	0.020406	NEK1,STAU1,LOC102170024,ARID1B,ADAM10,RALGAPB,ORC1,NEDD4,SNW1,UNC13C,SETMAR,VWDE,GLDC,RBL1,ATL1,DSG3,C6H4orf22,SPATA6L,LIPC,SUMF1,ZFYVE9,TRERF1,CLEC16A,CDC37L1,KDM4C,AK3,IFNLR1,CPN1,RNF24,CDH16,GPC3,CRADD,DNAJC18,RBM39,PIGK,LUZP6,LOC102186029,DNMBP,RCL1,ZNF280D,COX2,CIITA,FARP1,SH3TC2,SAMHD1,FRMD4A,PRPF18,BBS5,CDK19,LOC102169849,PTPRS,RNF103,IL22RA1,ANK2
GO:0042301	phosphate ion binding	0.022209	MTMR7,DNMBP,FASTKD1,CRADD
GO:0044422	organelle part	0.022558	GPC3,CDH16,DNAJC18,CRADD,RBM39,PIGK,DNMBP,RCL1,LUZP6,LOC102186029,COX2,ZNF280D,CIITA,SH3TC2,FARP1,SAMHD1,PRPF18,FRMD4A,BBS5,LOC102169849,CDK19,PTPRS,ANK2,RNF103,IL22RA1,NEK1,STAU1,LOC102170024,RALGAPB,ADAM10,ARID1B,ORC1,NEDD4,SETMAR,UNC13C,SNW1,ATL1,GLDC,VWDE,RBL1,C6H4orf22,DSG3,SPATA6L,LIPC,CLEC16A,ZFYVE9,TRERF1,SUMF1,AK3,KDM4C,CDC37L1,RNF24,IFNLR1,CPN1
GO:0007005	mitochondrion organization	0.024697	LOC102169849,CDC37L1,KDM4C,SETMAR,GLDC,ATL1,C6H4orf22,ADAM10
GO:0031647	regulation of protein stability	0.024746	MTMR7,FASTKD1,CRADD,DNMBP
GO:0050821	protein stabilization	0.024746	CRADD,FASTKD1,DNMBP,MTMR7
GO:0006886	intracellular protein transport	0.025781	LOC102169849,MBNL3,CDC37L1,KDM4C,UHRF2,IL22RA1,TRIM6,ANK2,FRMD4A,MCM8,CSE1L,

			CHMP3,ATL1,C6H4orf22,NEDD4,FARP1,UNC13C,GPC3,NEK1,LOC102170024,CRADD,ND5,ADAM10,MTMR7,FRMPD3,PIGK,LOC102179840
GO:0033644	host cell membrane	0.026113	ARID1B,KDM4C,ATP11C,UNC13C,SUMF1,ZNF280D,CDK19,LOC102169849
GO:0044218	other organism cell membrane	0.026113	CDK19,LOC102169849,ZNF280D,SUMF1,KDM4C,ATP11C,UNC13C,ARID1B
GO:0044279	other organism membrane	0.026113	CDK19,LOC102169849,ZNF280D,SUMF1,ATP11C,KDM4C,UNC13C,ARID1B
GO:0044765	single-organism transport	0.026249	DSG2,TRERF1,SUMF1,LIPC,UHRF2,KDM4C,MROH8,LOC102175219,AK3,CDC37L1,ATL1,MCM8,CHMP3,GLDC,SPATA6L,C6H4orf22,NEDD4,UNC13C,ND5,LOC102170024,STAU1,NEK1,ADIG,SLC23A1,MTPN,FRMPD3,ADAM10,ARID1B,LOC102169849,SOGA1,MBNL3,CDK19,ACAP1,ANK2,RNF103,PTPRS,FRMD4A,PRPF18,KDM4B,COX2,ZNF280D,LUZP6,ATP6,SH3TC2,FARP1,CIITA,DNAJC18,PDP2,CRADD,SLC12A4,GPC3,PIGK,LOC102179840
GO:0016559	peroxisome fission	0.026295	SAMHD1,RNF24
GO:0009396	folic acid-containing compound biosynthetic process	0.026838	ARID1B,NEK1,GPC3
GO:0007530	sex determination	0.027154	PAIP2,CLEC16A
GO:0007531	mating type determination	0.027154	PAIP2,CLEC16A
GO:0007532	regulation of mating-type specific transcription, DNA-dependent	0.027154	CLEC16A,PAIP2
GO:0045165	cell fate commitment	0.027154	PAIP2,CLEC16A
GO:0045895	positive regulation of mating-type specific transcription, DNA-dependent	0.027154	PAIP2,CLEC16A
GO:0006083	acetate metabolic process	0.027814	CRADD,ORC1
GO:0007031	peroxisome organization	0.029875	LOC102169849,SAMHD1,GPC3,RNF24,MCF2
GO:0016998	cell wall macromolecule catabolic process	0.031072	MBNL3,KDM4C,ADAM10
GO:0008374	O-acyltransferase activity	0.031748	SLC12A4,LOC102169849,LCAT
GO:0009986	cell surface	0.032255	CLEC16A,LOC102169849
GO:0006997	nucleus organization	0.032311	FARP1,KDM4C,LOC102169849
GO:0051260	protein homooligomerization	0.032568	PRND,KCTD11,PRNP
GO:0001948	glycoprotein binding	0.032948	ATL1,GPC3
GO:0043394	proteoglycan binding	0.032948	GPC3,ATL1
GO:0043395	heparan sulfate proteoglycan binding	0.032948	GPC3,ATL1
GO:0004861	cyclin-dependent protein serine/threonine kinase inhibitor	0.032954	FRMD4A,UNC13C

	activity		
GO:0030682	evasion or tolerance of host defense response	0.034321	GPC3,UNC13C,KDM4C,ADAM10
GO:0051807	evasion or tolerance of defense response of other organism involved in symbiotic interaction	0.034321	UNC13C,ADAM10,KDM4C,GPC3
GO:0032940	secretion by cell	0.034581	CHMP3,C6H4orf22,ARID1B,ADAM10,PIGK,LOC102169849,CDK19,CIITA,UNC13C
GO:0046903	secretion	0.036339	PIGK,C6H4orf22,ADAM10,ARID1B,CHMP3,UNC13C,CIITA,CDK19,LOC102169849
GO:0004375	glycine dehydrogenase (decarboxylating) activity	0.037124	GLDC
GO:0016642	oxidoreductase activity, acting on the CH-NH2 group of donors, disulfide as acceptor	0.037124	GLDC
GO:0015031	protein transport	0.038395	PIGK,LOC102179840,MTMR7,ARID1B,ADAM10,FRMPD3,ND5,CRADD,LOC102170024,GPC3,NEK1,UNC13C,FARP1,CIITA,NEDD4,C6H4orf22,ATL1,CSE1L,MCM8,CHMP3,FRMD4A,TRIM6,ANK2,IL22RA1,UHRF2,KDM4C,CDC37L1,PTPRS,ZFYVE9,MBNL3,LOC102169849
GO:0045184	establishment of protein localization	0.038395	NEDD4,CIITA,FARP1,UNC13C,NEK1,GPC3,LOC102170024,CRADD,ND5,MTMR7,FRMPD3,ADAM10,ARID1B,LOC102179840,PIGK,LOC102169849,ZFYVE9,MBNL3,CDC37L1,PTPRS,KDM4C,IL22RA1,UHRF2,ANK2,TRIM6,FRMD4A,CSE1L,MCM8,CHMP3,ATL1,C6H4orf22
GO:0006164	purine nucleotide biosynthetic process	0.038542	AK3,KDM4C,RNF103,LIPC,LOC102169849,TRERF1,TMEM106B,C6H4orf22,PANK2,ATL1,RBL1,CIITA,UNC13C,ATP6,COX2,MTPN,MTMR7,PIGK,NEK1,GPC3
GO:0010628	positive regulation of gene expression	0.039132	RCL1,LIPC,CLEC16A,MBNL3,UNC13C,LOC102170024,C6H4orf22,ORC1,PAIP2
GO:0045893	positive regulation of transcription, DNA-dependent	0.039132	UNC13C,RCL1,LIPC,CLEC16A,MBNL3,C6H4orf22,ORC1,PAIP2,LOC102170024
GO:0045935	positive regulation of nucleobase-containing compound metabolic process	0.039132	LOC102170024,C6H4orf22,PAIP2,ORC1,RCL1,LIPC,MBNL3,CLEC16A,UNC13C
GO:0051173	positive regulation of nitrogen compound metabolic process	0.039132	PAIP2,ORC1,C6H4orf22,LOC102170024,UNC13C,CLEC16A,MBNL3,LIPC,RCL1
GO:0051254	positive regulation of RNA metabolic process	0.039132	C6H4orf22,ORC1,PAIP2,LOC102170024,UNC13C,LIPC,RCL1,CLEC16A,MBNL3
GO:0051259	protein oligomerization	0.03958	C6H4orf22,ARID1B,KCTD11,PRND,RCL1,PRNP

GO:0016337	cell-cell adhesion	0.04075	ZFYVE9,DSG2,LOC102169849,CDH16,DSG3
GO:0009891	positive regulation of biosynthetic process	0.040756	UNC13C,CLEC16A,MBNL3,LIPC,RCL1,ORC1,PAIP2,C6H4orf22,LOC102170024
GO:0010557	positive regulation of macromolecule biosynthetic process	0.040756	UNC13C,LIPC,RCL1,CLEC16A,MBNL3,C6H4orf22,PAIP2,ORC1,LOC102170024
GO:0031328	positive regulation of cellular biosynthetic process	0.040756	UNC13C,LIPC,RCL1,CLEC16A,MBNL3,C6H4orf22,PAIP2,ORC1,LOC102170024
GO:0034613	cellular protein localization	0.04104	FRMD4A,MCM8,CSE1L,CHMP3,ATL1,C6H4orf22,MBNL3,LOC102169849,CDC37L1,KDM4C,UHRF2,IL22RA1,ANK2,TRIM6,GPC3,NEK1,CRADD,LOC102170024,ND5,MTMR7,ADAM10,FRMPD3,RALGAPB,LOC102179840,PIGK,NEDD4,UNC13C,FARP1
GO:0070727	cellular macromolecule localization	0.04104	UHRF2,IL22RA1,TRIM6,ANK2,CDC37L1,KDM4C,LOC102169849,MBNL3,C6H4orf22,CHMP3,MCM8,CSE1L,ATL1,FRMD4A,FARP1,UNC13C,NEDD4,LOC102179840,PIGK,FRMPD3,MTMR7,ADAM10,RALGAPB,LOC102170024,CRADD,ND5,NEK1,GPC3
GO:0005777	peroxisome	0.04117	RNF24,MCF2,LOC102169849,SAMHD1,GPC3
GO:0042579	microbody	0.04117	MCF2,RNF24,LOC102169849,GPC3,SAMHD1
GO:0019062	viral attachment to host cell	0.041326	ATP11C,GPC3,SAMHD1,LOC102169849,ZNF280D
GO:0044650	adhesion of symbiont to host cell	0.041326	ATP11C,GPC3,SAMHD1,LOC102169849,ZNF280D
GO:0043231	intracellular membrane-bounded organelle	0.042239	CIITA,FARP1,SH3TC2,LUZP6,DNMBP,ZNF280D,COX2,PIGK,GPC3,NFS1,DNAJC18,PDP2,PTPRS,BBS5,LOC102169849,MBNL3,PRPF18,FRMD4A,SNW1,SLC23A1,LOC102170024,CDC37L1,RBL2,KDM4C,AK3,CPN1,LOC102179449,LIPC,SUMF1,CLEC16A,TRERF1,DSG3,MCF2,CHMP3,RBL1,GLDC,ATL1,LOC102175415,LOC102186029,RCL1,RBM39,LOC102179840,CHGB,CRADD,IL22RA1,RNF103,ANK2,CDK19,SOGA1,SAMHD1,BPI,UNC13C,SETMAR,NEDD4,ARID1B,FRMPD3,ADAM10,RALGAPB,ORC1,NEK1,STAU1,IFNLR1,RNF24,ZFYVE9,C6H4orf22,PAIP2,SPATA6L,VWDE
GO:0072522	purine-containing compound biosynthetic process	0.043432	MTPN,MTMR7,PIGK,GPC3,NEK1,CIITA,UNC13C,ATP6,COX2,C6H4orf22,PANK2,ATL1,RBL1,AK3,KDM4C,RNF103,LIPC,LOC102169849,TRERF1,TMEM106B
GO:0043227	membrane-bounded organelle	0.043577	PRPF18,FRMD4A,LOC102169849,MBNL3,BBS5,PTPRS,DNAJC18,PDP2,NFS1,GPC3,PIGK,COX2,Z

			NF280D,DNMBP,LUZP6,SH3TC2,FARP1,CIITA,ATL1,CHMP3,GLDC,RBL1,MCF2,DSG3,TRERF1,CLEC16A,SUMF1,LIPC,LOC102179449,CPN1,KDM4C,AK3,RBL2,CDC37L1,LOC102170024,SLC23A1,SNW1,SAMHD1,BPI,SOGA1,CDK19,ANK2,IL22RA1,RNF103,CRADD,CHGB,LOC102179840,RBM39,RCL1,LOC102186029,LOC102175415,VWDE,PAIP2,SPATA6L,C6H4orf22,ZFYVE9,RNF24,IFNLR1,STAU1,NEK1,ORC1,RALGAPB,ADAM10,ARID1B,FRMPD3,NEDD4,SETMAR,UNC13C
GO:0050750	low-density lipoprotein particle receptor binding	0.044364	LOC102179840
GO:0070325	lipoprotein particle receptor binding	0.044364	LOC102179840
GO:0009306	protein secretion	0.044519	CHMP3,LOC102169849,UNC13C,PIGK,ADAM10,CIITA,ARID1B
GO:0009055	electron carrier activity	0.044737	ARID1B,RBL1,GLDC,NUTF2,DNAJC18,GPC3,TRIM34,UNC13C,CYTB,KDM4C,SUMF1,LOC102169849,SOGA1
GO:0030255	protein secretion by the type IV secretion system	0.045483	ADAM10,CHMP3
GO:0044097	secretion by the type IV secretion system	0.045483	ADAM10,CHMP3
GO:0005739	mitochondrion	0.045617	FRMD4A,GLDC,ATL1,DSG3,C6H4orf22,SPATA6L,LIPC,SUMF1,TRERF1,LOC102169849,CDC37L1,KDM4C,AK3,RNF103,NEK1,GPC3,LOC102170024,ADAM10,RALGAPB,ORC1,PIGK,DNMBP,COX2,FARP1,UNC13C,LOC102175415,SETMAR
GO:1901293	nucleoside phosphate biosynthetic process	0.045647	GPC3,NEK1,PIGK,MTPN,MTMR7,COX2,ATP6,UNC13C,CIITA,ATL1,RBL1,GLDC,PANK2,C6H4orf22,LOC102169849,TRERF1,TMEM106B,LIPC,RNF103,KDM4C,AK3
GO:0010921	regulation of phosphatase activity	0.045762	LOC102184370
GO:0035303	regulation of dephosphorylation	0.045762	LOC102184370
GO:0043666	regulation of phosphoprotein phosphatase activity	0.045762	LOC102184370
GO:0006611	protein export from nucleus	0.047147	FARP1,LOC102169849
GO:0006998	nuclear envelope organization	0.047147	FARP1,LOC102169849
GO:0005186	pheromone activity	0.048394	ZNF280D,CLEC16A,ARID1B,PAIP2
GO:0044406	adhesion to host	0.049731	ATP11C,GPC3,SAMHD1,ZNF280D,LOC102169849
GO:0051825	adhesion to other organism involved in symbiotic interaction	0.049731	ATP11C,GPC3,SAMHD1,ZNF280D,LOC102169849

Regions of low heterozygosity and high genetic differentiation from DBG			
**GO_accession**	**Description**	**Over_represented _pValue**	**Gene_names**
GO:0051260	protein homooligomerization	0.00060344	KCTD11,CACNB2,KCTD10,KCTD19,PRND
GO:0051259	protein oligomerization	0.0036155	PTPRN2,CACNB2,PRND,KCTD19,DENND1A,KCTD11,CNTN5,KCTD10
GO:0008277	regulation of G-protein coupled receptor protein signaling pathway	0.004154	LOC102173128,LRRC36,IQGAP1,MYCBP2
GO:0005518	collagen binding	0.013735	FAT3
GO:0008140	cAMP response element binding protein binding	0.015249	CACNB2
GO:0051289	protein homotetramerization	0.015249	CACNB2
GO:0005672	transcription factor TFIIA complex	0.017561	GPC3,IGSF21,PTPRN2
GO:0016668	oxidoreductase activity, acting on a sulfur group of donors, NAD(P) as acceptor	0.018019	IQGAP1,PTPRN2,CNTN5,KDM3A
GO:0047134	protein-disulfide reductase activity	0.018019	CNTN5,KDM3A,PTPRN2,IQGAP1
GO:0044036	cell wall macromolecule metabolic process	0.018095	DENND1A,MBNL3,ADAM10,PTPRN2
GO:0032505	reproduction of a single-celled organism	0.021904	CGNL1,CCDC64,TSC22D3
GO:0043093	cytokinesis by binary fission	0.021904	TSC22D3,CCDC64,CGNL1
GO:0000917	barrier septum assembly	0.02207	MYCBP2,TSC22D3,CCDC64,CGNL1
GO:0032506	cytokinetic process	0.02207	CGNL1,TSC22D3,CCDC64,MYCBP2
GO:0090529	cell septum assembly	0.02207	MYCBP2,TSC22D3,CCDC64,CGNL1
GO:0005876	spindle microtubule	0.022814	CGNL1,RALGAPB
GO:0009521	photosystem	0.023603	PRDM2,GPC3,MBNL3,RBL1,GCN1L1,ANK2,MYCBP2,CCDC64,WARS2,FAT3,KDM1A,FASTKD1,CACNB2,SH3TC2,PCBP3,DENND1A,CNTN5
GO:0016998	cell wall macromolecule catabolic process	0.024191	DENND1A,ADAM10,MBNL3
GO:0009055	electron carrier activity	0.02518	RBL1,DIP2C,DENND1A,DNAJC18,GPC3,SACM1L,WARS2,CNTN5,STARD13,FAT3,GCN1L1,MYCBP2,SOGA1
GO:0009085	lysine biosynthetic process	0.025225	CNTN5,MYO1H
GO:0009089	lysine biosynthetic process via diaminopimelate	0.025225	CNTN5,MYO1H
GO:0046451	diaminopimelate metabolic process	0.025225	MYO1H,CNTN5
GO:0004864	protein phosphatase inhibitor activity	0.027665	LOC102184370,FAT3
GO:0019212	phosphatase inhibitor activity	0.027665	FAT3,LOC102184370

GO:0009539	photosystem II reaction center	0.028236	CACNB2,RBL1,SH3TC2,DENND1A,GPC3,WARS2,CNTN5,FAT3,ANK2,GCN1L1,CCDC64
GO:0000045	autophagic vacuole assembly	0.030221	SPATA5,CNTN5
GO:0016236	macroautophagy	0.030221	SPATA5,CNTN5
GO:0007031	peroxisome organization	0.030269	CNTN5,PAIP2B,FAT3,SAMHD1,GPC3
GO:0016559	peroxisome fission	0.031533	FAT3,SAMHD1
GO:0019012	virion	0.032487	DIP2C,ZMYND11,PTPRN2,IGSF21,TSC22D3,GPC3,WARS2,RB1CC1,FAT3,MYCBP2,UBE3B,DENND1A,DIP2A,PLEKHG4,CNTN5,PXN,SPATA5,MSI1,ADAM10,PRDM2,MYO1H,ATP6V0D1,LOC102189985,ANK2,CCDC64,ECSCR,KDM3A,PCBP3,MROH8,ATP11C,SAMHD1,LOC102178382
GO:0003910	DNA ligase (ATP) activity	0.034183	FAT3,PCBP3
GO:0015926	glucosidase activity	0.035618	LOC102171901,MYCBP2,PTPRN2
GO:0003909	DNA ligase activity	0.036488	FAT3,PCBP3,IGSF21
GO:0010921	regulation of phosphatase activity	0.036949	LOC102184370
GO:0035303	regulation of dephosphorylation	0.036949	LOC102184370
GO:0043666	regulation of phosphoprotein phosphatase activity	0.036949	LOC102184370
GO:0003006	developmental process involved in reproduction	0.037166	LOC102186320,IGSF21,PAIP2,CNTN5,SIRT4
GO:0071554	cell wall organization or biogenesis	0.038662	CGNL1,DENND1A,PTPRN2,MBNL3,ADAM10
GO:0019904	protein domain specific binding	0.038933	LRP2,WARS2
GO:0006553	lysine metabolic process	0.039135	MYO1H,CNTN5
GO:0005777	peroxisome	0.039936	CNTN5,PAIP2B,FAT3,SAMHD1,GPC3
GO:0042579	microbody	0.039936	CNTN5,PAIP2B,FAT3,SAMHD1,GPC3
GO:0007033	vacuole organization	0.042177	SPATA5,CNTN5
GO:0009579	thylakoid	0.042776	FASTKD1,KDM1A,DENND1A,PCBP3,SH3TC2,CACNB2,SOGA1,CNTN5,GPC3,MBNL3,PRDM2,DIP2C,RBL1,CCDC64,MYCBP2,ANK2,GCN1L1,FAT3,WARS2
GO:0044436	thylakoid part	0.042776	MYCBP2,GCN1L1,ANK2,CCDC64,WARS2,FAT3,PRDM2,GPC3,MBNL3,RBL1,DIP2C,SOGA1,CNTN5,KDM1A,FASTKD1,SH3TC2,CACNB2,PCBP3,DENND1A
GO:0030683	evasion or tolerance by virus of host immune response	0.046176	ADAM10,GPC3,CNTN5
GO:0015556	C4-dicarboxylate transmembrane transporter activity	0.046925	ANK2
GO:0006461	protein complex assembly	0.048657	KCTD10,CNTN5,KCTD11,SOGA1,ANK2,KCTD19,PRND,PCBP3,DIP2C,DENND1A,PAIP2B,CACNB2,CGNL1,GPC3,PTPRN2

GO:0070271	protein complex biogenesis	0.048657	KCTD11,SOGA1,ANK2,KCTD10,CNTN5,GPC3,PTPRN2,PRND,KCTD19,PCBP3,DENND1A,DIP2C,PAIP2B,CGNL1,CACNB2
**Regions of low heterozygosity and high genetic differentiation from IMCG**			
**GO_accession**	**Description**	**Over_represented_pValue**	**Gene_names**
GO:0016413	O-acetyltransferase activity	0.0011868	PARD3B,CELF2,RFX7
GO:0004814	arginine-tRNA ligase activity	0.0030288	PRTG,CELF2,PHACTR4,DACH2
GO:0006420	arginyl-tRNA aminoacylation	0.0030288	PHACTR4,PRTG,CELF2,DACH2
GO:0017150	tRNA dihydrouridine synthase activity	0.0046779	ST3GAL1,UNC13C
GO:0044030	regulation of DNA methylation	0.0060874	PIGK,CELF2
GO:0016226	iron-sulfur cluster assembly	0.0068692	LOC102180113,KDM4C,PARD3B,CIAPIN1,UNC13C
GO:0031163	metallo-sulfur cluster assembly	0.0068692	KDM4C,LOC102180113,PARD3B,UNC13C,CIAPIN1
GO:0007276	gamete generation	0.0073418	SBF1,PRTG,PARD3B,KDM4C,FAT1,DMD,TPPP3,DACH2
GO:0005185	neurohypophyseal hormone activity	0.007363	KDM4C,RYBP,FARP1,CELF2
GO:0042287	MHC protein binding	0.0074953	PRTG,PARD3B,DMD,CRB1
GO:0042289	MHC class II protein binding	0.0074953	CRB1,PRTG,PARD3B,DMD
GO:0048609	multicellular organismal reproductive process	0.0087266	DMD,DACH2,TPPP3,PARD3B,SBF1,PRTG,FAT1,KDM4C,ETV5
GO:0032504	multicellular organism reproduction	0.0088716	DMD,DACH2,TPPP3,PARD3B,PRTG,SBF1,FAT1,KDM4C,ETV5
GO:0006997	nucleus organization	0.0094508	FAT1,KDM4C,PARD3B,FARP1
GO:0031145	anaphase-promoting complex-dependent proteasomal ubiquitin-dependent protein catabolic process	0.010049	PARD3B,GIGYF2
GO:0003896	DNA primase activity	0.010972	ST3GAL1,ZNF280D,DMD,KDM4C,FARP1,GIGYF2,RYBP,UNC13C
GO:0002064	epithelial cell development	0.014677	PARD3B,FAT1,KDM4C
GO:0007281	germ cell development	0.014677	FAT1,KDM4C,PARD3B
GO:0007286	spermatid development	0.014677	PARD3B,FAT1,KDM4C
GO:0007289	spermatid nucleus differentiation	0.014677	PARD3B,FAT1,KDM4C
GO:0035092	sperm chromatin condensation	0.014677	PARD3B,FAT1,KDM4C
GO:0048515	spermatid differentiation	0.014677	PARD3B,FAT1,KDM4C
GO:0007283	spermatogenesis	0.014892	FAT1,KDM4C,TPPP3,SBF1,PARD3B
GO:0048232	male gamete generation	0.014892	SBF1,PARD3B,TPPP3,KDM4C,FAT1
GO:0004308	exo-alpha-sialidase activity	0.015089	PARD3B,TTC23,UNC13C

GO:0016997	alpha-sialidase activity	0.015089	PARD3B,TTC23,UNC13C
GO:0048193	Golgi vesicle transport	0.015905	UNC13C,DDAH1,PARD3B,CELF2,RFX7,ST3GAL1,SEC23B,C6H4orf22,DACH2,NEDD4,PIGK,LOC102190889,DMD
GO:0006305	DNA alkylation	0.017961	C6H4orf22,PIGK,CELF2
GO:0006306	DNA methylation	0.017961	PIGK,CELF2,C6H4orf22
GO:0044728	DNA methylation or demethylation	0.017961	C6H4orf22,CELF2,PIGK
GO:0008374	O-acyltransferase activity	0.018866	PARD3B,CELF2,RFX7
GO:0044702	single organism reproductive process	0.019274	PARD3B,SBF1,PRTG,FAT1,KDM4C,DMD,DACH2,TPPP3
GO:0016973	poly(A)+ mRNA export from nucleus	0.021212	STAU1,DACH2,DMD,LOC102190889
GO:0051382	kinetochore assembly	0.021273	PARD3B,CHUK,UNC13C
GO:0051383	kinetochore organization	0.021273	UNC13C,PARD3B,CHUK
GO:0019953	sexual reproduction	0.021312	KDM4C,FAT1,PARD3B,PRTG,SBF1,TPPP3,CCDC136,DACH2,DMD
GO:0044085	cellular component biogenesis	0.021336	TTC23,PRND,FARP1,GIGYF2,CELF2,SETMAR,ZBTB16,CIAPIN1,INO80,LOC102179840,CHUK,HMBOX1,PRNP,DMD,C6H4orf22,LOC102180113,DACH2,UNC13C,PRTG,PARD3B,LOC102173932,KDM4C,SYNM,LOC102190889,MCF2L2,LOC102181499
GO:0022607	cellular component assembly	0.022445	ZBTB16,TTC23,PRND,FARP1,CELF2,SETMAR,LOC102180113,C6H4orf22,DACH2,CIAPIN1,INO80,LOC102179840,CHUK,PRNP,HMBOX1,DMD,LOC102173932,KDM4C,SYNM,UNC13C,PRTG,PARD3B,MCF2L2,LOC102190889
GO:0006406	mRNA export from nucleus	0.022532	DACH2,STAU1,DMD,LOC102190889,FARP1
GO:0030855	epithelial cell differentiation	0.022553	FAT1,KDM4C,PARD3B
GO:0060429	epithelium development	0.022553	KDM4C,FAT1,PARD3B
GO:0005643	nuclear pore	0.023244	STAU1,DACH2,MCF2L2,CCDC136,DMD,LOC102190889
GO:0006742	NADP catabolic process	0.023298	POLR2C
GO:0009109	coenzyme catabolic process	0.023298	POLR2C
GO:0019364	pyridine nucleotide catabolic process	0.023298	POLR2C
GO:0072526	pyridine-containing compound catabolic process	0.023298	POLR2C
GO:0044723	single-organism carbohydrate metabolic process	0.025744	HMBOX1,PHACTR4,CHUK,AKTIP,IFNLR1,C6H4orf22,FARP1,SUMF1,COQ9,SETMAR,PEX1,ZBTB16,FLNC,UBR4,RBL2,CWF19L1,DDAH1,PARD3B,ETV5,MIOX,DMD,CCDC136,DACH2,RYBP,TTC23,FAT1,NEDD4,LOC102176507,GATAD1,ST3GAL1,MCF2L2,UNC13C,CRB1,LOC102181835,KDM4C,

			SYNM
GO:0003922	GMP synthase (glutamine-hydrolyzing) activity	0.026391	PARD3B,DMD,CELF2,GIGYF2,UNC13C
GO:0006177	GMP biosynthetic process	0.026391	CELF2,DMD,PARD3B,UNC13C,GIGYF2
GO:0046037	GMP metabolic process	0.026391	GIGYF2,UNC13C,PARD3B,DMD,CELF2
GO:0009200	deoxyribonucleoside triphosphate metabolic process	0.026408	MCF2L2,UNC13C,DMD
GO:0009211	pyrimidine deoxyribonucleoside triphosphate metabolic process	0.026408	MCF2L2,UNC13C,DMD
GO:0046080	dUTP metabolic process	0.026408	MCF2L2,UNC13C,DMD
GO:1901137	carbohydrate derivative biosynthetic process	0.027249	MCF2L2,ST3GAL1,GATAD1,LOC102176507,NEDD4,SYNM,POLR3F,KDM4C,LOC102181835,CRB1,UNC13C,DACH2,CCDC136,DMD,FAT1,CELF2,AK3,TTC23,RYBP,GIGYF2,UBR4,FLNC,PIGK,ETV5,PARD3B,DDAH1,CWF19L1,RBL2,C6H4orf22,IFNLR1,AKTIP,CHUK,PHACTR4,ZBTB16,PEX1,SUMF1,COQ9,FARP1
GO:0033645	host cell endomembrane system	0.02812	EMC1,KDM4C
GO:0044165	host cell endoplasmic reticulum	0.02812	EMC1,KDM4C
GO:0044167	host cell endoplasmic reticulum membrane	0.02812	KDM4C,EMC1
GO:0044385	integral to membrane of host cell	0.02812	EMC1,KDM4C
GO:0044386	integral to host endoplasmic reticulum membrane	0.02812	KDM4C,EMC1
GO:0022884	macromolecule transmembrane transporter activity	0.028338	KDM4C,DACH2,UNC13C
GO:0016043	cellular component organization	0.02953	PRNP,DMD,CDC37L1,CCDC136,DACH2,CELF2,GIGYF2,TTC23,FAT1,LOC102190889,MCF2L2,SEC23B,PRTG,UNC13C,SYNM,KDM4C,HMBOX1,PHACTR4,CHUK,LOC102179840,INO80,CIAPIN1,ERLIN1,C6H4orf22,LOC102180113,SETMAR,SENP5,RFX7,FARP1,PRND,PEX1,ZBTB16,CSE1L,PARD3B,DDAH1,LOC102173932
GO:1901135	carbohydrate derivative metabolic process	0.02999	KDM4C,SYNM,POLR3F,UNC13C,CRB1,LOC102181835,ST3GAL1,MCF2L2,NEDD4,LOC102176507,GATAD1,ZNF280D,FAT1,RYBP,GIGYF2,TTC23,PYCR1,AK3,CELF2,CCDC136,DACH2,DMD,ETV5,RBL2,CWF19L1,DDAH1,PARD3B,UBR4,FLNC,PIGK,PEX1,ZBTB16,COQ9,SUMF1,FARP1,AKTIP,IFNLR1,C6H4orf22,PHACTR4,CHUK
GO:0005635	nuclear envelope	0.030685	DMD,LOC102190889,FARP1,DACH2,MCF2L2,CCDC136,STAU1
GO:0005741	mitochondrial outer membrane	0.032387	RFX7,DMD,PIGK,PARD3B,CDC37L1,C6H4orf22,K

			DM4C
GO:0031968	organelle outer membrane	0.032387	PIGK,DMD,PARD3B,RFX7,CDC37L1,KDM4C,C6H4orf22
GO:1901070	guanosine-containing compound biosynthetic process	0.032388	UNC13C,GIGYF2,PARD3B,DMD,CELF2
GO:0005102	receptor binding	0.032401	STAU1,UBR4,MCF2L2,DOK4,FGF5,LOC102176507,ZNF280D,LOC102190889,ETV5,KDM4C,LOC102173932,SETD3,CRB1,DDAH1,UNC13C,PRTG,PARD3B,C6H4orf22,DACH2,CCDC136,LOC102179840,ELOVL2,HMBOX1,DMD,ZBTB16,FARP1,RYBP,CELF2
GO:0046930	pore complex	0.033272	MCF2L2,DACH2,CCDC136,STAU1,LOC102190889,DMD
GO:0006955	immune response	0.033748	PRTG,PARD3B,CRB1,SETD3,ZNF280D,HMBOX1,DMD,LOC102179840,DACH2,CCDC136,STAU1,UBR4
GO:0003899	DNA-directed RNA polymerase activity	0.034028	F13B,DACH2,DMD,SNW1,ZBTB16,GIGYF2,FARP1,RYBP,UBR4,ST3GAL1,ZNF280D,POLR3F,KDM4C,POLR2C,PRTG,UNC13C
GO:0033644	host cell membrane	0.035194	UNC13C,SUMF1,PARD3B,EMC1,KDM4C,LOC102176507,DMD,ZNF280D
GO:0044218	other organism cell membrane	0.035194	SUMF1,UNC13C,PARD3B,EMC1,KDM4C,LOC102176507,DMD,ZNF280D
GO:0044279	other organism membrane	0.035194	DMD,ZNF280D,LOC102176507,KDM4C,PARD3B,EMC1,SUMF1,UNC13C
GO:0016482	cytoplasmic transport	0.03621	CDC37L1,PIGK,LOC102190889,DMD,STAU1,C6H4orf22,ST3GAL1,SEC23B,DACH2,DDAH1,FARP1,RFX7,PARD3B,KDM4C
GO:0071840	cellular component organization or biogenesis	0.036261	LOC102190889,MCF2L2,SEC23B,PRTG,UNC13C,SYNM,KDM4C,PRNP,DMD,CDC37L1,CCDC136,DACH2,CELF2,GIGYF2,TTC23,FAT1,LOC102181499,CSE1L,PARD3B,DDAH1,LOC102173932,HMBOX1,CHUK,PHACTR4,LOC102179840,CIAPIN1,INO80,ERLIN1,LOC102180113,C6H4orf22,SETMAR,RFX7,SENP5,FARP1,PRND,PEX1,ZBTB16
GO:0009236	cobalamin biosynthetic process	0.037201	CWF19L1,CRB1,CCDC136,SETD3
GO:0009147	pyrimidine nucleoside triphosphate metabolic process	0.037524	DMD,UNC13C,MCF2L2
GO:0006486	protein glycosylation	0.038635	CHUK,PHACTR4,DMD,C6H4orf22,IFNLR1,AKTIP,DACH2,TTC23,RYBP,SUMF1,FARP1,COQ9,ZBTB16,FAT1,LOC102176507,NEDD4,GATAD1,FLNC,ST3GAL1,UBR4,MCF2L2,CRB1,DDAH1,CWF19L1,UNC13C,RBL2,LOC102181835,PARD3B,ETV5,K

			DM4C,SYNM
GO:0009101	glycoprotein biosynthetic process	0.038635	AKTIP,IFNLR1,C6H4orf22,DACH2,DMD,CHUK,PHACTR4,ZBTB16,FAT1,RYBP,SUMF1,COQ9,FARP1,TTC23,ST3GAL1,UBR4,MCF2L2,NEDD4,LOC102176507,GATAD1,FLNC,KDM4C,ETV5,SYNM,RBL2,UNC13C,CRB1,CWF19L1,DDAH1,PARD3B,LOC102181835
GO:0043413	macromolecule glycosylation	0.038635	NEDD4,LOC102176507,FLNC,GATAD1,UBR4,ST3GAL1,MCF2L2,UNC13C,RBL2,CRB1,CWF19L1,DDAH1,PARD3B,LOC102181835,KDM4C,ETV5,SYNM,DMD,CHUK,PHACTR4,AKTIP,C6H4orf22,IFNLR1,DACH2,SUMF1,RYBP,COQ9,FARP1,TTC23,ZBTB16,FAT1
GO:0003972	RNA ligase (ATP) activity	0.038985	RFX7,DMD
GO:0005133	interferon-gamma receptor binding	0.039354	DMD,HMBOX1
GO:0009100	glycoprotein metabolic process	0.039459	KDM4C,ETV5,SYNM,RBL2,UNC13C,CRB1,DDAH1,CWF19L1,PARD3B,LOC102181835,UBR4,ST3GAL1,MCF2L2,NEDD4,LOC102176507,FLNC,GATAD1,ZBTB16,FAT1,SUMF1,RYBP,FARP1,COQ9,TTC23,AKTIP,IFNLR1,C6H4orf22,DACH2,DMD,CHUK,PHACTR4
GO:0070085	glycosylation	0.040076	LOC102181835,PARD3B,CWF19L1,CRB1,DDAH1,RBL2,UNC13C,SYNM,ETV5,KDM4C,GATAD1,FLNC,LOC102176507,NEDD4,MCF2L2,UBR4,ST3GAL1,TTC23,SUMF1,RYBP,COQ9,FARP1,FAT1,ZBTB16,PHACTR4,CHUK,DMD,DACH2,C6H4orf22,IFNLR1,AKTIP
GO:0008817	cob(I)yrinic acid a,c-diamide adenosyltransferase activity	0.040896	CWF19L1
GO:0016884	carbon-nitrogen ligase activity, with glutamine as amido-N-donor	0.041372	CELF2,PARD3B,DMD,GIGYF2,UNC13C
GO:0071822	protein complex subunit organization	0.041794	FARP1,TTC23,PRND,SETMAR,RFX7,INO80,PRNP,DMD,CHUK,C6H4orf22,DACH2,UNC13C,DDAH1,PARD3B,PRTG,LOC102173932,LOC102190889,MCF2L2
GO:0006405	RNA export from nucleus	0.042315	FARP1,LOC102190889,DMD,STAU1,DACH2
GO:0051168	nuclear export	0.042315	DACH2,STAU1,LOC102190889,DMD,FARP1
GO:0005184	neuropeptide hormone activity	0.043172	KDM4C,CRB1,FARP1,RYBP,PRTG,CELF2,LOC102190889
GO:0043414	macromolecule methylation	0.04363	SETD3,C6H4orf22,DMD,PIGK,PHACTR4,CELF2,UNC13C
GO:0022414	reproductive process	0.044401	PARD3B,SBF1,PRTG,UNC13C,FAT1,KDM4C,ETV5,DMD,DACH2,TPPP3

GO:0022412	cellular process involved in reproduction in multicellular organism	0.046042	FAT1,DACH2,KDM4C,PARD3B
GO:0051260	protein homooligomerization	0.047174	PRNP,DMD,PRND
GO:0009235	cobalamin metabolic process	0.047336	CCDC136,SETD3,CWF19L1,CRB1
GO:0016757	transferase activity, transferring glycosyl groups	0.047874	NEDD4,LOC102176507,GATAD1,ST3GAL1,MCF2L2,UNC13C,CRB1,LOC102181835,KDM4C,SYNM,DMD,CCDC136,DACH2,RYBP,GIGYF2,TTC23,FAT1,FLNC,UBR4,RBL2,DDAH1,CWF19L1,PARD3B,ETV5,CHUK,PHACTR4,AKTIP,IFNLR1,C6H4orf22,SUMF1,FARP1,COQ9,PEX1,LFNG
GO:0008194	UDP-glycosyltransferase activity	0.047995	LOC102181835,PARD3B,DDAH1,CWF19L1,CRB1,UNC13C,RBL2,SYNM,ETV5,KDM4C,GATAD1,FLNC,LOC102176507,NEDD4,ST3GAL1,UBR4,TTC23,FARP1,SUMF1,RYBP,COQ9,FAT1,PEX1,PHACTR4,CHUK,DMD,DACH2,CCDC136,IFNLR1,C6H4orf22,AKTIP
GO:0015399	primary active transmembrane transporter activity	0.049297	IQCE,POLR3F,RFX7,UNC13C,CRB1,CCDC136,C6H4orf22,DMD,HMBOX1
GO:0015405	P-P-bond-hydrolysis-driven transmembrane transporter activity	0.049297	HMBOX1,DMD,CCDC136,C6H4orf22,RFX7,CRB1,UNC13C,POLR3F,IQCE
GO:0006535	cysteine biosynthetic process from serine	0.049754	RFX7
GO:0009001	serine O-acetyltransferase activity	0.049754	RFX7
GO:0016412	serine O-acyltransferase activity	0.049754	RFX7
GO:0019344	cysteine biosynthetic process	0.049754	RFX7
GO:0007091	metaphase/anaphase transition of mitotic cell cycle	0.049924	GIGYF2,DMD,PARD3B
GO:0030071	regulation of mitotic metaphase/anaphase transition	0.049924	GIGYF2,PARD3B,DMD
GO:0044772	mitotic cell cycle phase transition	0.049924	GIGYF2,PARD3B,DMD
GO:0044784	metaphase/anaphase transition of cell cycle	0.049924	PARD3B,DMD,GIGYF2
GO:1901990	regulation of mitotic cell cycle phase transition	0.049924	GIGYF2,DMD,PARD3B
GO:1902099	regulation of metaphase/anaphase transition of cell cycle	0.049924	DMD,PARD3B,GIGYF2

Supplementary table 7 KEGG-pathways enriched for genes affected by selections

Alleles frequency difference of DBG			
**ID**	**Term**	**P-Value**	**Input**
chx04014	Ras signaling pathway	0.005267	RASGRP2|RRAS|PLA2G1B|KDR|IKBKG
chx04120	Ubiquitin mediated proteolysis	0.005343	STUB1|MID2|LOC102174728|BIRC6

chx04130	SNARE interactions in vesicular transport	0.013342	VAMP8|VAMP5
chx05205	Proteoglycans in cancer	0.019978	RRAS|ANK2|PXN|KDR
chx04623	Cytosolic DNA-sensing pathway	0.030621	IRF3|IKBKG
chx04370	VEGF signaling pathway	0.033703	PXN|KDR
chx04010	MAPK signaling pathway	0.036023	RASGRP2|RRAS|PTPN7|IKBKG
chx04622	RIG-I-like receptor signaling pathway	0.048342	IRF3|IKBKG
chx04920	Adipocytokine signaling pathway	0.049549	IKBKG|LOC102190823
**Alleles frequency difference of IMCG**			
**ID**	**Term**	**P-Value**	**Input**
chx00480	Glutathione metabolism	0.008585	LOC102188087|IDH1
chx00310	Lysine degradation	0.009929	TAB1|LOC102170078
chx04810	Regulation of actin cytoskeleton	0.022618	RRAS|GIT1|PIKFYVE
chx01210	2-Oxocarboxylic acid metabolism	0.048023	IDH1
chx00604	Glycosphingolipid biosynthesis - ganglio series	0.048023	SLC33A1
**Genetic differentiation**			
**ID**	**Term**	**P-Value**	**Input**
chx00190	Oxidative phosphorylation	7.33E-13	LOC102179840|ATP8|COX2|COX3|CYTB|COX1|ATP6|ND4L|ND1|ND3|ND2|ND5|ND4|ND6
chx05012	Parkinson's disease	1.35E-12	LOC102179840|ATP8|COX2|COX3|CYTB|COX1|ATP6|ND4L|ND1|ND3|ND2|ND5|ND4|ND6
chx01100	Metabolic pathways	6.55E-05	LOC102179840|PANK2|LOC102178836|COX2|COX3|CYTB|COX1|ATP6|NFS1|ND4L|ATP8|LOC102171894|GLDC|ND1|ND3|ND2|ND5|ND4|ND6
chx05010	Alzheimer's disease	0.000799	ATP8|COX2|COX3|CYTB|COX1|ATP6
chx05016	Huntington's disease	0.001187	ATP8|COX2|COX3|CYTB|COX1|ATP6
chx04260	Cardiac muscle contraction	0.001282	COX2|COX3|CYTB|COX1
chx00770	Pantothenate and CoA biosynthesis	0.005166	PANK2|LOC102171894
**Regions of low heterozygosity and high genetic differentiation from DBG**			
**ID**	**Term**	**P-Value**	**Input**
chx03010	Ribosome	0.003873	LOC102178382|RPLP0|LOC102181054|LOC102173128|MROH8|LOC102190542
chx00565	Ether lipid metabolism	0.025395	PLD2|PLA2G1B
chx04120	Ubiquitin mediated proteolysis	0.035203	UBE3B|LOC102174728|MID2
chx04122	Sulfur relay system	0.049279	NFS1

Regions of low heterozygosity and high genetic differentiation from IMCG

ID	Term	P-Value	Corrected P-Value
chx03020	RNA polymerase	0.004583	0.265798
chx03010	Ribosome	0.015456	0.303012
chx00230	Purine metabolism	0.020767	0.303012
chx01230	Biosynthesis of amino acids	0.026822	0.303012
chx05169	Epstein-Barr virus infection	0.027301	0.303012
chx00240	Pyrimidine metabolism	0.042239	0.303012

29
